# Structural Alterations in a Component of Cytochrome *c* Oxidase and Molecular Evolution of Pathogenic *Neisseria* in Humans

**DOI:** 10.1371/journal.ppat.1001055

**Published:** 2010-08-19

**Authors:** Marina Aspholm, Finn Erik Aas, Odile B. Harrison, Diana Quinn, Åshild Vik, Raimonda Viburiene, Tone Tønjum, James Moir, Martin C. J. Maiden, Michael Koomey

**Affiliations:** 1 Department of Molecular Biosciences, University of Oslo, Oslo, Norway; 2 Centre for Molecular Biology and Neuroscience, University of Oslo, Oslo, Norway; 3 Department of Zoology, University of Oxford, Oxford, United Kingdom; 4 Department of Biology (Area 10), University of York, Heslington, York, United Kingdom; 5 Institute of Microbiology, University of Oslo, Oslo, Norway; Northwestern University Feinberg School of Medicine, United States of America

## Abstract

Three closely related bacterial species within the genus *Neisseria* are of importance to human disease and health. *Neisseria meningitidis* is a major cause of meningitis, while *Neisseria gonorrhoeae* is the agent of the sexually transmitted disease gonorrhea and *Neisseria lactamica* is a common, harmless commensal of children. Comparative genomics have yet to yield clear insights into which factors dictate the unique host-parasite relationships exhibited by each since, as a group, they display remarkable conservation at the levels of nucleotide sequence, gene content and synteny. Here, we discovered two rare alterations in the gene encoding the CcoP protein component of cytochrome *cbb*_3_ oxidase that are phylogenetically informative. One is a single nucleotide polymorphism resulting in CcoP truncation that acts as a molecular signature for the species *N. meningitidis*. We go on to show that the ancestral *ccoP* gene arose by a unique gene duplication and fusion event and is specifically and completely distributed within species of the genus *Neisseria*. Surprisingly, we found that strains engineered to express either of the two CcoP forms conditionally differed in their capacity to support nitrite-dependent, microaerobic growth mediated by NirK, a nitrite reductase. Thus, we propose that changes in CcoP domain architecture and ensuing alterations in function are key traits in successive, adaptive radiations within these metapopulations. These findings provide a dramatic example of how rare changes in core metabolic proteins can be connected to significant macroevolutionary shifts. They also show how evolutionary change at the molecular level can be linked to metabolic innovation and its reversal as well as demonstrating how genotype can be used to infer alterations of the fitness landscape within a single host.

## Author Summary

The closely related bacterial species *N. meningitidis*, *N. gonorrhoeae* and *N. lactamica* exclusively colonise mucosal surfaces in humans. While *N. gonorrhoeae* leads to gonorrhea, the other two species persist mainly in their host in the absence of disease. *N. meningitidis* does occasionally cause severe, life threatening illness, however. Little is known about the factors and elements that dictate the unique human interactions exhibited by each species. Moreover, the evolutionary relationships between these species are poorly characterized. Here, we describe two successive alterations in a single gene that can be linked first to all species within the genus *Neisseria* and then the species *N. meningitidis*. We also show these signature alterations have phenotypic consequences by affecting core respiratory metabolic processes. These findings have significant implications for the evolution of related bacterial species within a single host and provide a novel perspective on the episodic and reversible nature of innovative adaptation.

## Introduction

The genus *Neisseria* comprises Gram-negative, oxidase-positive diplococci that are frequently isolated from the mucosal surfaces of humans and two closely related species are important pathogens of man [1]. *Neisseria gonorrhoeae* is the etiologic agent of gonorrhea that remains one of the most common sexually transmitted diseases contributing to worldwide morbidity, mortality and infertility. Although treatable with antibiotics, no vaccine is currently available against the gonococcus. *Neisseria meningitidis* is primarily a commensal of the human oropharynx that, under incompletely understood circumstances, causes invasive disease and meningitis. Most cases of meningococcal disease are caused by clonal complexes of related sequence types (STs), the so-called hyper-invasive lineages [2]. These lineages are underrepresented in healthy carriers and significant numbers of individuals are colonized with carriage isolates belonging to an array of STs that rarely cause disease [3].

Despite their differing host interactions, mechanisms of transmission and ecology, *N. gonorrhoeae* and *N. meningitidis* display remarkable conservation and uniformity at the levels of coding sequences, gene content and synteny. Nonetheless, comparative genome analyses have identified genes and gene clusters unique to either *N. gonorrhoeae* or *N. meningitidis* but few if any of the corresponding products can be specifically connected to the differential host interactions observed. A prime example of this situation would be the genes required for biosynthesis of polysaccharide capsule, which is essential to systemic meningococcal disease, and that are absent in *N. gonorrhoeae*. However, only a limited subset of capsular serogroups is associated with disease and 16–20% of meningococcal carriage isolates do not possess these genes [4]. A recent study documented that the presence of the insertion sequence IS1655 is restricted to *N. meningitidis* but how this element might relate to speciation or specifically to meningococcal biology remains unclear [5]. Attempts to reconcile the relationships between genotype and lifestyle of *N. meningitidis* and *N. gonorrhoeae* are further complicated by the existence of the closely related species *Neisseria lactamica*, a harmless commensal found predominantly in the upper respiratory tracts of infants and children [6]. In contrast to *N. meningitidis* in which carriage is low during infancy and rises to high levels in adolescents and young adults, carriage of *N. lactamica* is high in young children but declines with age [7]. Microarray-based genome hybridization studies showed that the majority of coding sequences are highly conserved in all three species although some genes unique to both *N. gonorrhoeae* and *N. meningitidis* were identified [8], [9], [10]. Included in the latter are the *iga1* and *pptA* genes encoding an extracellular endopeptidase and a protein targeting phosphoethanolamine transferase [11], [12]. Validation of the impact of putative virulence components has been hampered by a lack of relevant animal models for neisserial disease.

The highly conserved genetic structure and human host restriction observed for *N. gonorrhoeae* and *N. meningitidis* are most consistent with allopatric divergence from a single common ancestor. Such a model was first proposed by Vazquez and colleagues based on the relatively reduced diversity of *N. gonorrhoeae* strains measured by multilocus enzyme electrophoresis (vs *N. meningitidis*) of house keeping genes and the fact that the primary niche for all human *Neisseria* species other than *N. gonorrhoeae* is the oropharynx [13]. Specifically, it was suggested that *N. gonorrhoeae* arose as a clone of *N. meningitidis* that could colonize the urogenital tract. Prolonged physical isolation, niche specialization and genetic isolation would thus have driven speciation. This model was further supported by the analyses of the *porA* gene (encoding the class 1 outer membrane porin protein PorA) that is found in all strains of *N. meningitidis* and *N. gonorrhoeae* but absent in *N. lactamica* and other commensal *Neisseria* species [14]. While PorA is a major constituent of the outer membrane of most meningococcal isolates, all strains of *N. gonorrhoeae* examined to date carry an identical frameshift mutation that disrupts the integrity of the *porA* ORF [15]. Two other genes unique to the pathogenic *Neisseria*, *ggt* and *adhC* (encoding gamma-glutamyl transpeptidase and S-nitrosoglutathione oxidoreductase respectively), are also intact in *N. meningitidis* but inactivated due to frameshift mutations in *N. gonorrhoeae* [16], [17]. While the presence of these three pseudogenes in *N. gonorrhoeae* denotes descent from an organism carrying active forms of the genes, it remains unknown how extant isolates of *N. meningitidis* and *N. gonorrhoeae* might relate to such an ancestral population. Despite the potentially confounding contributions of shared ancestry and genetic exchange, a recent study utilizing multilocus sequence typing (MLST) demonstrated the ability to readily categorize isolates of *N. meningitidis*, *N. gonorrhoeae* and *N. lactamica* into three distinct species [18].

**Figure 1:**
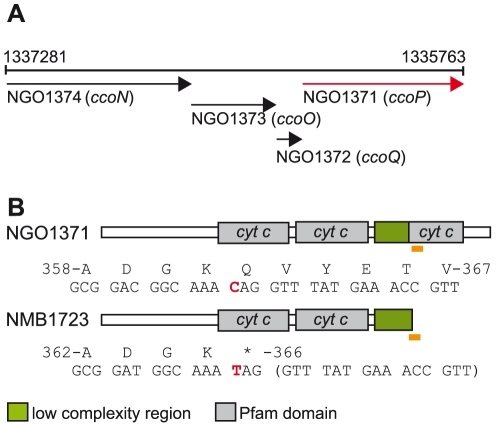
Organization of the neisserial *ccoNOQP* locus and domain architectures of *c*-type multiheme CcoPs. (A) The organization of the neisserial *ccoNOQP* gene cluster. Numbers denote the nucleotide sequence coordinates from the genome sequence of *N. gonorrhoeae* strain FA1090 (Genbank AE004969). (B) Modular structures of the cognate neisserial tri-heme CcoP and the di-heme form associated with isolates of *N. meningitidis*. The region encompassing the premature stop codon resulting in CcoP truncation and the corresponding region in tri-heme CcoP are indicated by yellow rectangles and the specific residues and the corresponding codons are numbered according to those of intact polypeptides. The single base change creating a premature stop codon in *ccoP_Nme_* (CAG to TAG) is indicated in red. Note that the codon involved corresponds to residue 366 in *N. meningitidis* but to residue 362 in gonococcal CcoP owing to a 12 base pair deletion in the *N. gonorrhoeae* alleles (Figure S1).

Oxygen reductase members of the heme-copper superfamily act as terminal oxidases in all domains of life and play a central role in aerobic energy generation and conservation [19]. Given their critical function, alterations in their structure are likely to have important consequences and therefore be targets of natural selection. Adaptive changes in cytochrome *c* oxidases have been implicated in major evolutionary transitions in anthropoid primates and carnivorous plants [20], [21]. Most bacteria utilize branched electron-transfer networks that enable them to use diverse electron donors and/or electron acceptors in respiration. The sole oxygen reductase catalysing the reduction of dioxygen to water encoded in *Neisseria* genomes is of the *c*-family or cytochrome *cbb*_3_ oxidase type [22]. Canonical cytochrome *cbb*_3_ oxidases consist of four subunits encoded by tandemly arranged genes (Figure 1A) [23]. CcoN is the highly conserved, catalytic subunit that contains a dinuclear centre formed by the iron of a high-spin heme and an associated copper ion (Cu_B_) where dioxygen is reduced [24]. CcoO and CcoP are both membrane-bound *c*-type cytochromes believed to channel electrons to the dinuclear center [25]. CcoQ is a small, single-spanning membrane protein believed to function in stabilizing the interaction of CcoP with the CcoNO complex [26]. The roles of CcoQ and CcoP may vary between species as active forms of the complex can be detected in their absence in some but not all cases [27], [28]. Cytochrome *cbb*_3_ oxidase in some organisms has a high affinity for oxygen and is often associated with growth under conditions of oxygen-restriction [22] and both *N. gonorrhoeae* and *N. meningitidis* inhabit niches associated with low oxygen tension [29], [30]. Importantly, cytochrome *cbb*_3_ oxidases couple oxygen reduction to translocating protons across the inner membrane such that metabolic energy is conserved for subsequent ATP synthesis [31].

*N. gonorrhoeae* and *N. meningitidis* also possess a truncated denitrification pathway in which nitrite (NO_2_^−^) is first reduced to nitric oxide (NO) by NirK (AniA) that is then reduced to nitrous oxide (N_2_O) by NorB [32], [33], [34], [35]. The linked *nirK* and *norB* genes are differentially controlled by a number of transcriptional regulators that are responsive to changes in the levels of oxygen, NO_2_^−^ and NO [36], [37], [38], [39], [40], [41]. Although previously considered to be an anaerobic process, denitrification under oxic conditions has been documented in many species including *N. gonorrhoeae and N. meningitidis* [35], [42]. Taken together, the latter species appear to be adapted to similar growth environments at distinct ecological sites (i.e. urogenital versus oropharyngeal mucosal sites) and have highly related respiratory chains that allow them to co-metabolize oxygen and nitrite as electron acceptors under microaerobic conditions.

A cardinal feature of *N. gonorrhoeae* and *N. meningitidis* is their highly recombinogenic nature that results from inter- and intraspecies genetic exchange [43]. Efficient lateral gene transfer in these instances is mediated through natural competence for transformation requiring specific uptake sequences in the donor DNA [44]. As these DNA uptake sequences are dramatically overrepresented only in the genomes of *Neisseria* species, this largely restricts imports to donors from within the genus. In the particular case of *N. meningitidis*, interspecies recombination at numerous loci encoding surface antigens has been widely documented [45], [46], [47]. Still as *N. gonorrhoeae*, *N. meningitidis* and *N. lactamica* species represent cohesive, differentiated entities [18], gene flow must be constrained at some level in order to limit convergence and despeciation [48]. However, the molecular and ecological factors constraining genetic structuring in these instances remain to be determined. Given these circumstances, the phylogenetic basis for the evolution of human *Neisseria* species remains poorly understood. Moreover, it remains unclear what genetic elements dictate the unique human interactions exhibited by each species.

Rare genomic changes, signature mutations occurring in the genomes of particular clades, have frequently been employed to resolve phylogenetic uncertainties [49]. Here, we report the identification of a single nucleotide polymorphism (SNP) that results in a molecular marker for the species *N. meningitidis* as it is found in all strains examined and absent from all isolates of *N. gonorrhoeae* and *N. lactamica* and of other commensals tested. Although this mutation results in the truncation of an essential component of the cytochrome *cbb*_3_ oxidase, the sole neisserial respiratory oxidase, it conditionally affects nitrite consumption by the nitrite reductase that functions in the denitrification pathway. These findings provide evidence that an alteration in the circuitry of respiratory electron-transfer networks is associated with *N. meningitidis* speciation.

## Results

### A SNP leading to truncation of the CcoP subunit of cytochrome *cbb*_3_ oxidase is associated with the species *N. meningitidis*

In the course of characterizing a general *O*-linked protein glycosylation system in *N. gonorrhoeae* strain MS11 [50], we discovered that its CcoP is a glycoprotein possessing a tri-heme *c*-type cytochrome domain architecture as opposed to the di-heme form found in all other CcoP proteins (as annotated by possessing the IPR004678 domain of the InterPro database) (Figure 1B). When the status of *N. meningitidis* CcoP was examined using the genome sequences for 5 strains available, the nucleotide and deduced amino acid sequences were highly related (greater than 97%) to that in *N. gonorrhoeae* save for the presence of transitional substitution in codon 366 (CAG to TAG resulting in a premature stop codon). The *N. meningitidis* forms would then be predicted to be truncated at the end of the AlaSerPro-rich, low complexity region (LCR) that separates the second and third *c*-type heme domains and encompasses the glycan attachment site [50]. DNA sequencing of *ccoP* from 78 additional *N. meningitidis* isolates used in this study (Table S1) further supported the presence of the SNP seen in the genomic sequences resulting in CcoP truncation. In contrast, *ccoP* DNA sequencing and accessing genome sequencing projects for which *ccoP* data were available revealed the absence of the SNP from 26 *N. gonorrhoeae* strains, 13 *N. lactamica* strains and 11 commensal *Neisseria* strains encompassing 8 species (Table S2). Thus, the association of the SNP with the species *N. meningitidis* was complete. Furthermore, we conclude that the tri-heme encoding *ccoP* allele is ancestral to the *N. meningitidis* alleles.

**Figure 2:**
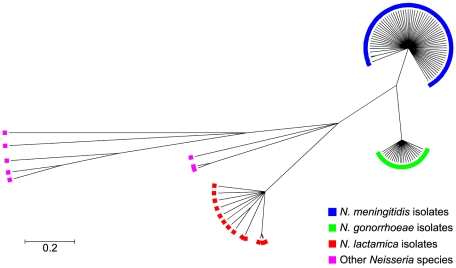
Genealogical representation of the *ccoP* gene among *Neisseria* strains using ClonalFrame. Phylogenetic trees were constructed using ClonalFrame version 1.1 available at http://www.xavierdidelot.xtreemhost.com/clonalframe.htm [74]. In the present study, over 300,000 iterations and 100,000 burn-ins were performed with every hundredth tree sampled after which, a 95% consensus tree was derived. Annotation was then undertaken by importing the tree into the Molecular Evolutionary Genetics Analysis software package (MEGA v4.0) [73].

Phylogenetic examination of *ccoP* genes using the majority rule consensus tree constructed with Clonalframe revealed five distinct branches corresponding to each of the *Neisseria* species: *N. meningitidis*, *N. gonorrhoeae*, *N. lactamica* and other *Neisseria* including *N. cinerea*, *N. polysaccharea*, *N. sicca*, *N. subflava* and *N. flavescens* further differentiating these isolates from one another. Within each clade, *ccoP* genes were well conserved (*p-*distances: *N. lactamica* = 0.010, *N. meningitidis* = 0.004, *N. gonorrhoeae* = 0.002) although *ccoP* genes belonging to commensal isolates other than *N. lactamica* were much more diverse (*p-*distance = 0.165) (Figure 2). This was apparent in the alignment of polymorphic sites (Figure S1) where synonymous and non-synonymous substitutions along the *ccoP* gene were more abundant when these neisserial commensals were included in the analysis further confirming the homology of *ccoP* genes belonging to *N. meningitidis*, *N. gonorrhoeae* and *N. lactamica* isolates. Recombination events among *ccoP* genes were uncommon with a total of five events predominantly among *ccoP* genes belonging to commensal *Neisseria*. One horizontal gene transfer event from a meningococcal isolate and gonococcal isolate to *N. cinerea* was also detected with high probability by ClonalFrame (Table S3).

### The extended, third *c*-type heme domain of CcoP impacts on denitrification respiratory flux

**Figure 3:**
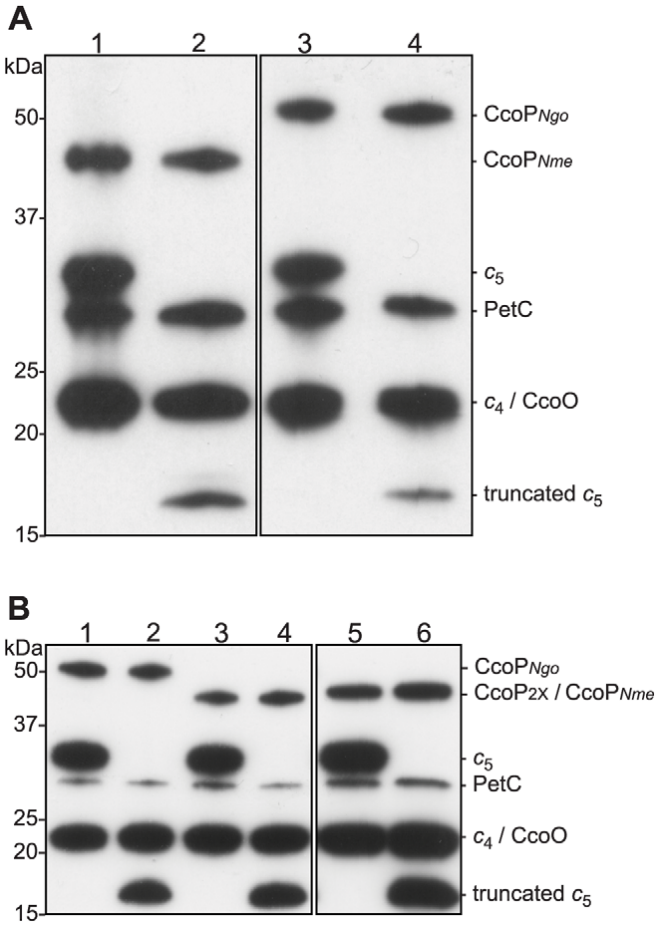
Heme-stained protein blots showing altered expression of *c*_5_ and CcoP in defined backgrounds. Samples of total cell extracts were separated by 12% SDS-PAGE, blotted and stained for heme-dependent peroxidase activity. (A) *N. meningitidis* strains: 1, wild type (MC58); 2, *cycB* (*c*_5_-); 3, *ccoP_Ngo_* (KS348); 4, *cycB*, *ccoP_Ngo_* (KS349). (B) *N. gonorrhoeae* strains: 1, wild-type (VD300); 2, *cycB* (KS336); 3, *ccoP*_2x_ (KS335); 4, *ccoP*_2x_, *cycB* (KS337); 5, *ccoP_Nme_* (KS340); 6, *ccoP_Nme_ cycB* (KS341).

Given the unique association between the SNP resulting in expression of the truncated, di-heme CcoP form and *N. meningitidis*, we sought to determine what phenotypic consequences might ensue from such an alteration. We therefore took a comparative approach to addressing this point by constructing strains of *N. meningitidis* MC58 expressing the tri-heme CcoP form found in *N. gonorrhoeae* and strains of *N. gonorrhoeae* VD300 expressing the truncated di-heme form from *N. meningitidis* (Figures 3A and B, respectively). This was done using transposon insertions mapping 3′ of *ccoP* as selectable markers in trans-species transformation experiments. Direct sequencing of *ccoP* was used to ensure that the entire, specific ORFs were transferred and exchanged in the recombinants used. In addition, a strain of *N. gonorrhoeae* was constructed in which solely the SNP responsible for CcoP truncation in *N. meningitidis* was incorporated into the otherwise unaltered *N. gonorrhoeae* allele. When subjected to a variety of aerobic growth conditions, we observed no differences between isogenic strains expressing the di- and tri-heme forms in either *N. meningitidis* or *N. gonorrhoeae* (Figures S2A, B and S3A, B respectively, data not shown). Thus, the skewed distribution of CcoP forms was not a consequence of gross metabolic incompatability as measured *in vitro*.

In another approach, BLAST searches were performed using the third heme encompassing domain immediately C-terminal of the major AlaSerPro-rich LCR in *N. gonorrhoeae* CcoP as a query. This segment was highly related to the C-terminally localized domain in cytochrome *c*_5_, a di-heme *c*-type cytochrome encoded by the *cycB* gene (NMB1677 in *N. meningitidis* and NGO1328 in *N. gonorrhoeae*). Like CcoP, *c*_5_ is predicted to be membrane-associated and also possesses an AlaSerPro-rich LCR that encompasses the attachment sites of its *O*-linked glycan between its two *c*-type heme domains [50]. In *N. meningitidis*, a tetracycline resistance gene cassette insertion mutation in *cycB* that disrupts the integrity of the ORF distal to the first heme domain was shown to abolish nitrite reduction and nitrite-dependent growth under microaerobic conditions [51]. In *N. gonorrhoeae*, a *cycB* null mutation was reported to result in increased sensitivity to growth inhibition by excess oxygen and small decreases in respiratory capacity [52]. Thus, *c*_5_ is a prime candidate to act as an electron carrier in pathways ultimately targeting both cytochrome *cbb*_3_ oxidase and NirK.

**Figure 4:**
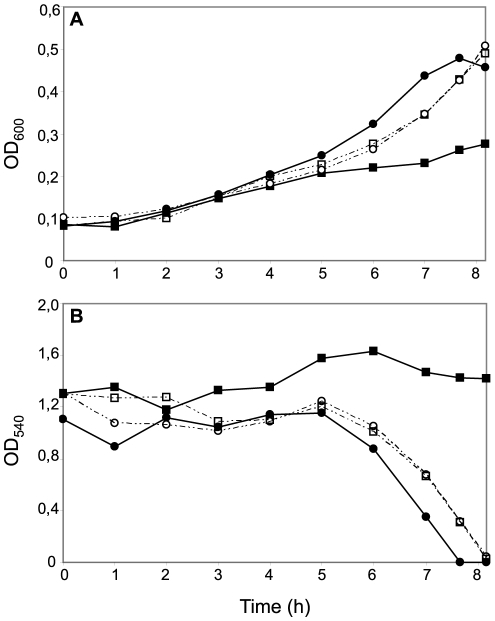
Effects of CcoP domain alterations on microaerobic growth and nitrite reduction in *N. meningitidis*. Cultures of wild-type (MC58) (open squares); *ccoP_Ngo_* (KS348) (open circles); *cycB* (*c*_5_-), (filled squares) and *ccoP_Ngo_*, *cycB* (KS349) (filled circles) growing under microaerobic conditions plus 5 mM nitrite (A). Growth was monitored by measuring OD_600_. (B) Nitrite reduction was monitored for microaerobic cultures from (A). The results shown are representative of three independent experiments.

Given the structural similarities between *c*_5_ and the tri-heme CcoP form, we examined what genetic interactions might exist between *cycB* and the different *ccoP* alleles with regard to the truncated dentrification pathway. To this end, a *cycB* null mutation was generated in which the entire open reading frame was deleted. Derivatives of *N. gonorrhoeae* carrying this allele were distinctive in that they exhibited a severe growth defect manifested as poor plating efficiency and slow growth that was not seen in equivalent strains carrying the previously characterized *cycB* insertion mutation (data not shown). While profiling of *c*-type heme proteins confirmed the absence of intact *c*_5_ in both backgrounds, strains carrying the *cycB* insertion mutation expressed a *c*-type heme protein whose migration corresponded to that predicted for a truncated, mono-heme *c*_5_ form resulting from disruption of the open reading frame at residue 171 (Figures 3A, B and Figure S4). As the *cycB* null mutation was pleiotropic, the *cycB* insertion mutation was used in this study. As previously reported [51], introduction of *cycB* insertion mutation into *N. meningitidis* MC58 led to a clear defect in nitrite-dependent, microaerobic growth and measurements from culture medium showed that this growth defect was associated with an inability to reduce nitrite (Figures 4A and B). In contrast, the strain carrying both the *ccoP_Ngo_* and *cycB* alleles was remarkably similar to the wildtype strain and that carrying only the *ccoP_Ngo_* allele in these phenotypes (Figures 4A and B). No differences in growth for any of these strains were seen under aerobic and microaerobic conditions (Figures S2A and B, data not shown). Thus, the *ccoP_Ngo_* allele was epistatic to *cycB* demonstrating that the CcoP tri-heme isoform and *c*_5_ overlap functionally in supporting NirK-mediated, nitrite reduction.

**Figure 5:**
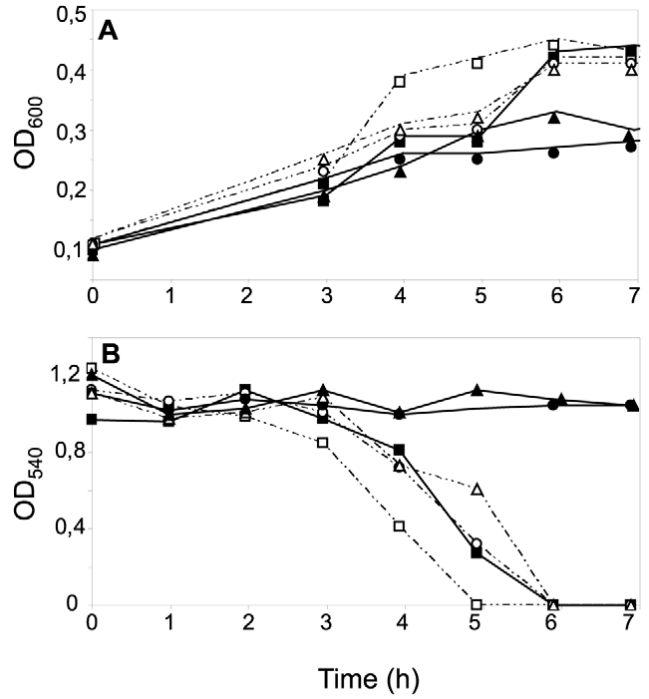
Effects of CcoP domain alterations on microaerobic growth and nitrite reduction in *N. gonorrhoeae*. Cultures of wild-type (VD300) (open squares); and mutants *ccoP*_2x_ (KS335) (open circles); *cycB* (KS336) (filled squares); *ccoP*_2x_, *cycB* (KS337) (filled circles); *ccoP_Nme_* (KS340) (open triangles); *ccoP_Nme_*, *cycB* (KS341) (filled triangles) growing under microaerobic conditions plus 5 mM nitrite (A). Growth was monitored by measuring OD_600_. (B) Nitrite reduction was monitored for microaerobic cultures from (A). The results shown are representative of three independent experiments.

Reciprocal experiments were carried out in *N. gonorrhoeae* VD300 using backgrounds carrying either endogenous *ccoP* or alleles encoding truncated di-heme CcoP forms. Here again, no significant differences in growth for any of these strains were seen under aerobic and microaerobic conditions (Figures S3A and B). Both *N. gonorrhoeae* strains expressing di-heme CcoP forms were reduced in rates of growth under nitrite supplemented, microaerobic conditions relative to the strain expressing the tri-heme form and this property was paralleled by delayed nitrite consumption (Figures 5A and B). These results were similar to those reported elsewhere in which missense mutations predicted to disrupt the integrity of the third *c*-type heme domain of *N. gonorrhoeae* CcoP were associated with diminished nitrite-dependent, microaerobic growth and reduced capacities to reduce nitrite [42]. These same phenotypes were exhibited by the *N. gonorrhoeae* strain with the *cycB* insertion mutation alone. However, when combined with the alleles encoding di-heme CcoP, the *cycB* mutation led to a complete defect in nitrite-dependent, microaerobic growth (Figure 5A). Measurements from culture medium showed that this growth defect was associated with the inability to reduce nitrite (Figure 5B). These phenotypes were not attributable to a defect in NirK expression, as the latter was detected at similar levels in all backgrounds (Figure S5). To ensure that the defect in nitrite reduction in these backgrounds was not due to a spurious mutation or genetic alteration arising during strain construction or propagation, a wildtype copy of *cycB* was reintroduced at an ectopic site. This led to restoration of nitrite-dependent, microaerobic growth and nitrite reduction in the *cycB*, *ccoP*_2X_ (di-heme form of *N. gonorrhoeae* CcoP made by changing codon number 362 from CAG to the stop codon TAG) backgrounds (data not shown). Taken together, these results document clear differences in the requirements for nitrite reduction associated with di- and tri-heme encoding *ccoP* alleles. Accordingly, the findings strongly suggest that strains of *N. meningitidis* are fundamentally distinct from those of other neisserial species with regard to respiratory denitrification.

### The di-heme CcoP form is essential to microaerobic growth in *N. meningitidis* and *N. gonorrhoeae*

**Figure 6:**
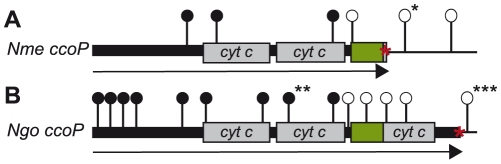
Distribution of *ccoP* transposon insertion mutations recovered in wildtype and merodiploid backgrounds. Shown are the sites of mTn insertions with open circles denoting those recoverable in *N. meningitidis* (A) and *N. gonorrhoeae* (B) wildtype backgrounds. Filled circles denote those that were only recoverable in strains carrying a second copy of *ccoP*. In the case of *N. meningitidis* merodiploid background in which both gene copies were active, insertions denoted by filled circles were recovered at both loci. In the case of *N. gonorrhoeae* merodiploid background, the second *ccoP* copy was inactive and all insertions denoted by filled circles mapped in the non-expressed copy. Symbols: arrows indicate the *ccoP* open reading frame, grey boxes represent regions encoding *c*-type heme domains, green boxes show AlaSerPro-rich, low complexity regions and red asterisks indicate stop codons. Insertions marked with single and triple black asterisks were used to swap *ccoP* alleles between *N. meningitidis* and *N. gonorrhoeae*. The insertion marked with double black asterisks was used to inactivate the endogenous *N. gonorrhoeae* allele in the background carrying the de-repressible *ccoP* allele at an ectopic site (see text).

To examine the role of CcoP in *N. meningitidis*, transposon insertion mutations were generated in the cloned *ccoP* gene that were then introduced into a wildtype background. However, transformants were only recovered for those insertions that mapped 3′ to the ORF encompassing the second *c*-type heme domain (i.e. within the LCR and third *c*-type heme domain). To ensure that this selectivity was not due to some peculiarity of the constructs themselves, a strain carrying a second active copy of *ccoP* at an ectopic site was created. Transformants for all insertion mutations were recovered using this merodiploid strain (Figure 6A). A similar approach was taken to examining CcoP function in *N. gonorrhoeae* with transposon insertion mutations being generated in the cloned gene that were then introduced into a wildtype background. As seen in the *N. meningitidis* wildtype background, transformants were only recovered for those mutations that mapped 3′ to the ORF encoding the second *c*-type heme domain (i.e. within the LCR and third *c*-type heme domain). Here as a control, a strain carrying a tandem duplication of *ccoP* (by Campbell-type plasmid integration) was used in which the distal gene copy was non-functional due to a deletion of the promoter and translational initiation sites. Transformants for all insertion mutations were recovered using this strain and importantly, all those that could not be recovered in the wildtype background mapped exclusively in the non-expressed *ccoP* copy (Figure 6B). As mutations that disrupt the integrity of the di-heme form are conditionally defective in microaerobic growth, CcoP appeared to be an essential gene product in both *N. meningitidis* and *N. gonorrhoeae*. Moreover, the structural features and constraints for minimal CcoP function (imparted by the di-heme form) were likely identical in both species.

**Figure 7:**
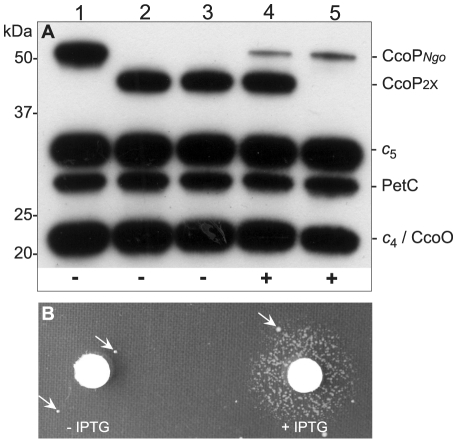
Conditional expression of CcoP confirms its essential role in growth. (A) Heme-stained blot of *N. gonorrhoeae* extracts showing profiles of *c*-type heme proteins. Total cell extracts from wildtype and mutants were separated by 10% SDS-PAGE, transferred to membrane and stained for heme-dependent peroxidase activity. Strains: 1, wildtype (KS101); 2, *ccoP*_2x_ (KS351); 3 and 4, *ccoP*_2x_, *ccoP*_ind_ (KS345); 5, *ccoP*_2x_::mTn*erm*26, *ccoP*_ind_ (KS346). CcoP*_Ngo_* and CcoP_2x_ mark the relative mobility of the tri-heme and di-heme forms. Note that strain *ccoP*_2x_::mTn*erm*26, *ccoP*_ind_ (lane 5) expresses only the inducible CcoP form. ‘+’ and ‘−’ at bottom denote the presence and absence of IPTG respectively in the culture media. As shown in (B), the strain expressing solely the inducible CcoP form requires IPTG for growth on solid medium. Arrows indicate the presence of suppressor mutants that are associated with IPTG-independent CcoP expression.

To confirm the essentiality of *ccoP*, a transcriptional fusion construct was made in which expression was linked to *tac*-*UV5* control sequences and introduced at an ectopic site in the neisserial genome. As this construct does not include an associated *lacI^Q^* gene, repression requires a background expressing LacI^Q^. For practical purposes, we therefore carried out these experiments in *N. gonorrhoeae* which enabled us to first introduce the ectopic de-repressible *ccoP* allele in a background expressing di-heme CcoP from the endogenous locus (Figure 7A). Transposon mutations previously established as abrogating CcoP function were then introduced into these backgrounds and transformants were selected in the presence of IPTG. Those that acquired IPTG dependent growth were readily identified in *N. gonorrhoeae* and in each instance, the transposon mapped to the endogenous *ccoP* gene (Figure 7B). It was quite easy to pick out revertants in which growth became IPTG-independent and in these instances, this phenotype was associated with restoration of de-regulated CcoP expression (data not shown). These findings demonstrated that CcoP was essential to growth under the microareobic conditions examined and reinforced the hypothesis that this effect was due to its role in cytochrome *cbb*_3_ oxidase activity.

### A *c*_5_-CcoP hybrid supports nitrite reduction and related phenotypes

**Figure 8:**
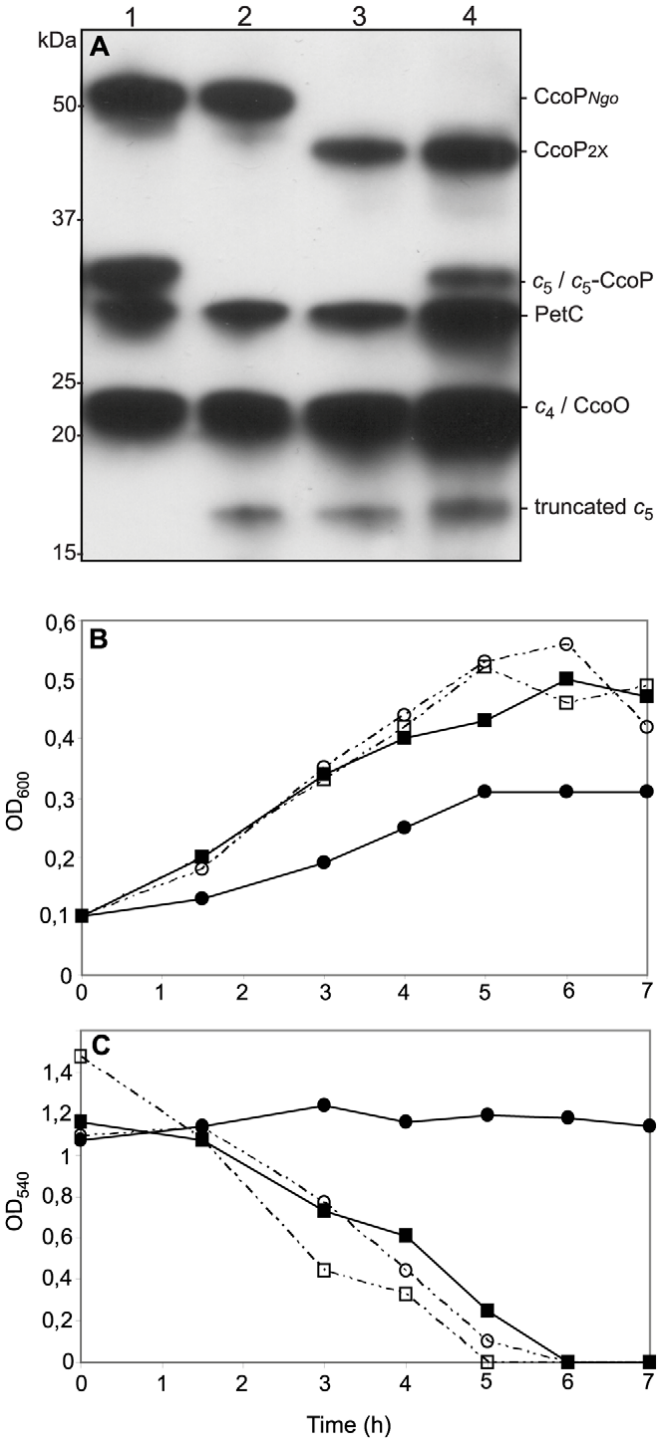
Ectopic expression of a *c*_5_-CcoP hybrid protein complements a defect in nitrite-dependent, microaerobic growth in *N. gonorrhoeae*. (A) Samples of total cell extracts from wildtype and mutants separated by SDS-PAGE, blotted and stained for *c*-type heme-dependent peroxidase activity. Strains: 1, wildtype (VD300); 2, *cycB* (KS336); 3, *cycB*, *ccoP*_2x_ (KS337); 4, *cycB*, *ccoP*_2x_, *cycB*-*ccoP* (KS347). (B) Cultures of wild-type (VD300) (open squares); and mutants *cycB* (KS336) (filled squares); *cycB*, *ccoP*_2x_ (KS337) (filled circles) and *cycB*, *ccoP*_2x_, *cycB*-*ccoP* (KS347) (open circles) growing under microaerobic conditions supplemented with 5 mM nitrite. (C) Nitrite depletion was monitored for microaerobic cultures from (B). The results shown are representative of three independent experiments.

The genetic interactions and degree of shared structural identity suggested that the C-terminal *c*-type heme domains of *c*_5_ and CcoP have related functions in chanelling electrons to support nitrite reduction activity. Therefore, a translational fusion consisting of the amino-terminus of *c*_5_ (encompassing the first *c*-type heme domain) and the third heme domain of *N. gonorrhoeae* CcoP was constructed. This was done in a manner such that the relative spacing between the two heme domains in the hybrid was maintained as seen for wildtype *c*_5_ and was facilitated by using a conserved stretch of residues in the AlaSerPro-rich linker domains (Figure S6). This gene fusion including the endogenous *cycB* promoter sequences was then introduced at an ectopic site in the *N. gonorrhoeae* background expressing the truncated *cycB* allele (from its endogenous site) and a di-heme expressing *ccoP* allele. The *c*_5_-CcoP chimaera was detected as a novel cytochrome *c* species that migrated with a mobility equivalent to that seen for endogenous *c*_5_ (Figure 8A). This hybrid gene restored both nitrite reduction and nitrite-dependent microareobic growth in this background (Figures 8B and C). These findings formally demonstrate the functional equivalence of the terminal heme domains of *N. gonorrhoeae* CcoP and *c*_5_ when displayed in the context of otherwise structurally identical polypeptides.

### A modular domain fusion with *c*_5_ accounts for the genesis of the tri-heme CcoP form

**Figure 9:**
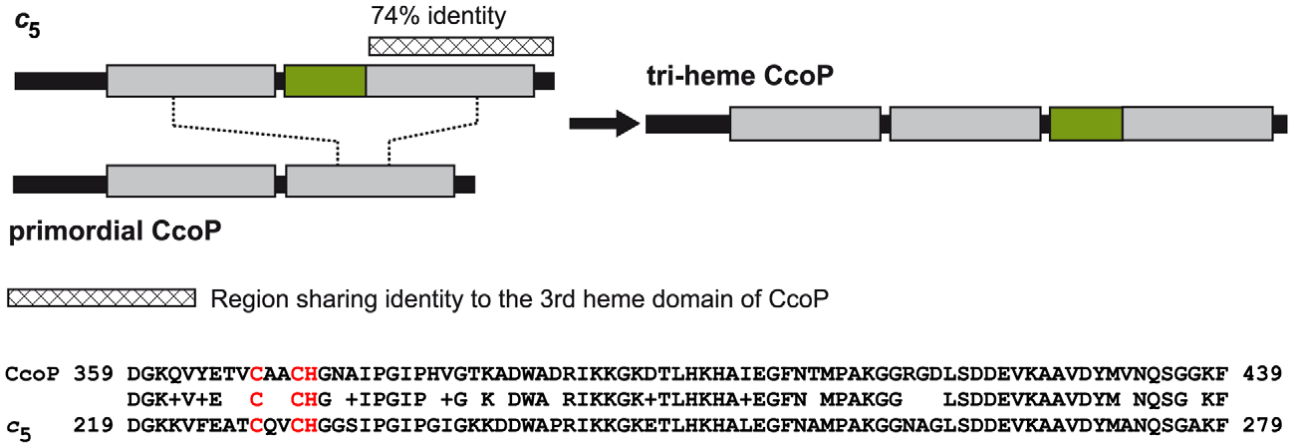
Model for the origin of the tri-heme CcoP in the genus *Neisseria*. Based on the shared domain architecture, high degrees of primary sequence identity and function, the most parsimonious model invokes a gene fusion event between *cycB* (encoding *c*_5_) and a primordial *c*-type di-heme encoding *ccoP* gene (top panel). This hypothesis is particularly well supported by the high degree of structural conservation between the carboxy-terminal segment (encompassing the third heme domain) of CcoP and the heme domains of *c*_5_ (heme domains in grey). Specifically, residues 359–439 of CcoP share 74% identity with the corresponding C-terminal segment of *c*_5_ and 47% with the first heme domain of *c*_5_ (bottom panel, the two cysteine and single histidine residues found in a CXXCH motif required for disulfide bonding to the vinyl groups of heme are shown in red). Secondly, such a scenario would account for the presence of the AlaSerPro-rich region (in green) between the second and third CcoP heme domains that is similar in sequence composition and length to the equivalent interdomain segment of *c*_5_. Finally, both of these same stretches each bear serine occupancy sites for the *O*-linked glycosylation [50].

Given its unusual activity and unique distribution within *Neisseria* species, the potential evolutionary origin of the tri-heme CcoP was examined. As the vast majority of CcoP forms identified to date are di-heme forms, a simple model might involve the amplification of the tandemly arrayed, heme encoding gene segments. However, this mechanism is inconsistent with the observed lack of nucleotide sequence identity encoding the spacer domains between the first and second heme units and the second and third heme units. Instead, the most parsimonious scenario entails a non-reciprocal recombination event involving the 3′ ends of the *c*_5_ gene and a primordial di-heme CcoP. In this model, unequal crossing over within the homologous sequences of the *c*_5_ di-heme - encoding and CcoP second heme - encoding segments would provide gene and protein structures most reconciliable with that of extant tri-heme CcoP forms (Figure 9). This hypothesis is particularly well supported by the high degree of structural conservation between the carboxy-terminal segment (encompassing the third heme domain) of CcoP and the two heme domains of *c*_5_. The last 81 residues of CcoP (359–439) share 74% sequence identity with the corresponding C-terminal segment of *c*_5_ and 47% with the first heme domain of *c*_5_. Such a scenario would account for the presence of the AlaSerPro-rich region between the second and third CcoP heme domains. Although it is difficult to establish amino acid identity within these AlaSerPro rich regions (due to their underlying low complexity), both the lengths of the AlaSerPro stretches (34 residues in *c*_5_ versus 42 in CcoP) and overall alanine richness (47.1% Ala residues in *c*_5_ versus 47.6% in CcoP) are similar between the two. Moreover, these stretches each bear serine occupancy sites for the glycans associated with the general *O*-linked glycosylation system in *N. gonorrhoeae* [50]. Taken together, the conserved structural features and shared functional attributes of *c*_5_ and the tri-heme CcoP provide strong evidence for modular-based evolution underlying CcoP neofunctionalization.

## Discussion

Three closely related species within the genus *Neisseria* represent an intriguing model system in which to investigate the evolution trajectories of related pathogenic and commensal bacteria in a single host. Here, we report the identification of a SNP unique in its association with the species *N. meningitidis* that leads to truncation of the *c*-type heme protein CcoP, an essential component of its sole, terminal respiratory cytochrome oxidase. The dichotomy in neisserial phylogeny revealed through this altered molecular character raises the obvious question as to why this change appears to be so adaptive on the one hand and yet at the same time, so restricted in its distribution. From the sole viewpoint of aerobic respiration, CcoP functions by supporting cytochrome *cbb*_3_ oxidase activity. However, we failed to discern any phenotypic alterations in either the *N. gonorrhoeae* or *N. meningitidis* strains varying solely in expression of the di- versus tri-heme forms under standard lab conditions. Therefore, a key factor here may be the altered circuitry of electron transfer favoring microaerobic (oxygen reduction by cytochrome *cbb*_3_ oxidase) versus combined microaerobic denitrifying (oxygen reduction by cytochrome *cbb*_3_ oxidase supplemented by nitrite reduction by NirK) respiration. For example, the ability of di-heme CcoP to promote solely cytochrome *cbb*_3_ oxidase activity (at the expense of reduced electron carriage to NirK) could be adaptive under more aerobic conditions or in situations where nitrite levels might be diminished. Moreover, nitrite reduction could come with significant metabolic cost as it generates nitric oxide that can be toxic and growth inhibitory [39]. Although *N. meningitidis* strains express the NorB nitric oxide reductase (reducing nitric oxide to innocuous nitrous oxide), toxic NO can accumulate so rapidly that growth inhibition occurs before sufficient NorB activity has been expressed (as it is specifically de-repressed by NO acting on the nitric oxide responsive repressor NsrR) [37], [39], [40]. This effect can be detected in *N. meningitidis* as NirK-dependent, nitrite sensitivity occurring under a number of growth conditions [53]. Additionally, there is strong evidence that *N. meningitidis* strains are under relaxed selective constraints with regard to NirK-mediated nitrite reduction. Specifically, *nirK* was not detected in 7 of 26 *N. meningitidis* strains examined by microarray-based genome hybridization technology [10]. Moreover, two reports examining *nirK* found frameshift and inactivating missense mutations in 5 of 23 and 8 of 31 strains respectively [54], [55]. Whilst the selection pressure for maintaining a functional NirK is relaxed in *N. meningitidis*, this is not the case in *N. gonorrhoeae* and other *Neisseria* species. In the latter strains, alleles encoding intact NirK are found in all available genomic and individual sequences (6 commensal neisserial strains and 36 strains of *N. gonorrhoeae* including those presented in Figure 2, Table S2 and in [54]. Thus, the fixation of the SNP- associated *ccoP* allele is consistent with the hypothesis that *N. meningitidis* is an organism in the process of evolving into a microaerobe that no longer supplements growth via NirK-mediated denitrification.

**Figure 10:**
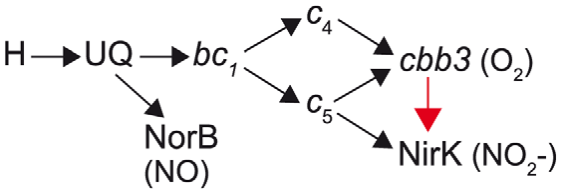
Proposed pathway for electron transfer from NADH to nitric oxide, oxygen and nitrite in *N. meningitidis* and *N. gonorrhoeae*. Electron flow from cytoplasmic reductants (NADH, succinate, etc. shown as [H]) to terminal electron acceptors NO, O_2_, and NO_2_ are shown by arrows. The unique pathway associated with the tri-heme CcoP form in *N. gonorrhoeae* (absent in *N. meningitidis*) is designated by the red arrow. UQ, ubiquinone; NirK, nitrite reductase; NorB, nitric oxide reductase. The model is based on data from this work and that found in [51], [52], [78].

Another extraordinary aspect of the *N. meningitidis ccoP* allele is the fact that it represents a reversal of a previous rare genetic change in CcoP domain architecture. We provide a data-based model for how the tri-heme encoding *ccoP* arose by a homologous recombination event between *c*_5_ and a primordial, di-heme encoding *ccoP* allele and suggest that like the case of the SNP mutation, this event occurred only once and radiated by vertical or lateral transmission due to its adaptive value. Thus, the *ccoP* tri-heme allele (in its wildtype and derivative SNP variant forms) is a molecular marker of species within the genus *Neisseria*. The ability of tri-heme CcoP to act as a redox partner in a pathway that ultimately can transfer electrons to NirK would dramatically reshape the organization of electron transport chain, providing broader connectivity to electrons emanating from *bc*_1_ through *c*_4_ as well as *c*_5_ (Figure 10) [51], [52]. Reprogramming of electron flow circuitry might facilitate fine-tuning of the partition of electrons between the oxygen and nitrite reduction pathways. Such a system might be particularly useful when both electron acceptors are available but vary dynamically in relative abundance. Further studies of *ccoP* status in neisserial species are warranted as the number of strains examined here was restricted and some findings may be subject to sample bias. This concern may be particularly relevant to the *N. meninigitidis* isolates carrying capsule null loci (*cnl*) and *N. lactamica* isolates used here that were derived from carriage studies within the United Kingdom as well to other commensal species for which the data set is small. Nonetheless, the *ccoP* tri-heme allele is present in all *Neisseria* species for which data are available and it is conspicuously absent in any other species including members of the most closely related genera in the family *Neisseriaceae* such as *Chromobacterium*, *Eikenella*, *Kingella*, and *Laribacter* and more distant relatives within *Betaproteobacteria*.

The SNP-associated *ccoP* allele suggests that *N. meningitidis* as a clade has either undergone a relatively recent, dramatic reduction in population size (with a *ccoP* SNP -bearing strain first to pass the bottleneck) or that there has been a selective sweep of the SNP allele through the population via natural genetic transformation. Both scenarios are plausible given the relatively short time period over which sequential bottlenecks associated with epidemic spread can lead to clonal descent [43], [56] and the well - established capacity for frequent intraspecies recombination [57], [58]. *N. meningitidis*, *N. lactamica* and other commensal *Neisseria* species inhabit the same apparent oropharyngeal niche and bi-directional, interspecific genetic exchange of some loci occurs frequently [59]. We assume therefore that strains of *N. meningitidis* expressing tri-heme CcoP and of *N. lactamica* and other commensal species expressing di-heme CcoP arise but are purged due to reduced fitness. Consistent with this idea, the low *p-*distances observed by ClonalFrame for *ccoP* genes belonging to *N. meningitidis*, *N. lactamica* and *N. gonorrhoeae* are indicative of the conserved nature within species such that diversity may result in reduced fitness of the organism. The lack of recombination observed by RDP3 analysis (Table S3) among *N. meningitidis*, *N. lactamica* and *N. gonorrhoeae* (when compared to those found in other *Neisseria* species which exhibited higher *p-*distance values) further support this hypothesis. Therefore, other as yet unidentified differences in gene repertoire or expression are likely epistatic to *ccoP*.

An important aspect of this topic relates to which redox partners carry out direct electron transfer to neisserial NirK. Although *in vitro* studies have identified both azurins (members of the cupredoxin family) and *c*-type heme cytochromes as electron donors to other members of the blue, copper - containing nitrite reductase (CuNIR) family [60], [61], only rarely have the physiological contributions been addressed *in vivo*. Although both *N. meningitidis* and *N. gonorrhoeae* express a lipoprotein form of azurin termed Laz, it has been reported that Laz is nonessential for growth under either aerobic or anerobic conditions (in the presence of nitrite) [62]. A null mutation in the gene encoding Cyt *c*_550_ protein was recently shown to disrupt NirK dependent growth and nitrite utilization in *Bradyrhizobium japonicum* [63]. Also, CuNIRs that carry *c*-type heme domains translationally fused to the carboxy-termini of the nitrite reductase domain have been identified in other proteobacterial species by genome mining [64]. Perhaps most relevant, the recent high-resolution crystal structure of a blue CuNIR together with a *c*-type cytochrome provided conclusive evidence for the direct transfer of electrons between these partners [65]. Based on the genetic interactions established between *cycB* and *ccoP* here along with related observations [42], we favor a model in which both of the corresponding gene products directly donate electrons to NirK. A prerequisite for such a model is that *c*_5_ and CcoP (presumably in the context of *cbb*_3_) come in close contact with NirK. However, the NirK lipoprotein in its active trimer form is proposed to be linked to the outer membrane [66], [67] while cytochrome *cbb*_3_ oxidases must be integrated into the inner membrane in order to fulfil their role in proton pumping essential to ATP synthesis. While the carboxy terminal extensions (encompassing both the flexible LCR and distal heme domain) found in CcoP and *c*_5_ might function to bridge a potential periplasmic gap, more detailed studies of the spacial distribution and organization of the structures involved are needed to address this matter. Regardless of the molecular nature of electron transfer between CcoP and NirK, our finding here that a component of the oxygen reducing respiratory complex can be essential to the activity of an alternative reductase is unprecedented.

In summary, these findings provide a novel perspective on the evolution of species within the genus *Neisseria*. They also demonstrate that microaerobic denitrification is a metabolic pathway of major influence in these bacteria and support the position that *N. gonorrhoeae*, *N. lactamica* and *N. meningitidis* fully deserve their designation as a distinct species, because they are clearly evolving independently. These findings also provide a dramatic example of how evolutionary change at the molecular level can be linked to metabolic innovation and its reversal as well as demonstrating how genotype can be used to infer alterations of the fitness landscape within a single host.

## Materials and Methods

### Bacterial strains, plasmids and growth conditions

A total of 71 *N. meningitidis* isolates initially employed in the evaluation of the (MultiLocus Sequence Typing) MLST typing method were used for *ccoP* sequence analysis [68]. The collection, which was assembled to be representative of organisms causing endemic and epidemic disease in the latter part of the 20^th^ century, included several isolates from each of seven recognised hyper-invasive clonal complexes: (Sequence Type) ST-1, ST-5, ST-4, ST-11, ST-32, ST-8, and ST-41/44 in addition, to isolates belonging to clonal complexes ST-22, ST-23 and ST-13 (cc269) as well as isolates with unique sequence types. A total of 11 unencapsulated *N. meningitidis* isolates containing the capsule null locus were also investigated and these included isolates from clonal complexes ST-53, ST-198 and ST-334 (Table S1). The non-pathogenic *Neisseria* and *N. gonorrhoeae* isolates used for sequence studies are listed in Table S2. All isolates described above were grown overnight on Mueller Hinton agar supplemented with 5% defibrillated sterile horse blood in a 5% CO_2_ atmosphere. DNA was extracted using an IsoQuick Nucleic Acid Extraction kit (Orca Research Inc.) according to manufacturer's instructions. The neisserial strains used for functional studies are described in Table 1 and unless otherwise stated, all these strains were cultured on conventional GC plates over night at 37°C, in the presence of 5% CO_2_ as described previously [69]. Aerobic cultures were carried out in 7.5 ml of broth in 50 ml tubes shaken at 190 r.p.m (SARSTEDT, Nümbrecht, D). Microaerobic culture was carried out using 23 ml of GC broth, supplemented with 10mM NaHCO_3_, in 25 ml Sterilin McCartney bottle shaken at 190 r.p.m. [35]. Where appropriate, cultures were supplemented with 5mM NaNO_2_. Growth was monitored by measuring the optical density at 600 nm (OD_600_) in a WPA biowave CO 8000 Cell Density Meter. *E. coli* DH5α and HB101 were used for plasmid propagation and cloning experiments and were grown on Luria-Bertani media (LB). Antibiotics were used at the following concentrations for *N. gonorrhoeae*: chloramphenicol, 10µg/ml; erythromycin, 8µg/ml; tetracycline, 4µg/ml; for *N. meningitidis*: erythromycin, 8µg/ml; tetracycline, 20µg/ml; for *E. coli*: chloramphenicol, 30 µg/ml; erythoromycin 300µg/ml; kanamycin 50µg/ml; ampicillin 100µg/ml; tetracycline, 15µg/ml; streptomycin, 100µg/ml. pUP6 is a derivative of pHSS6 that carries two gonococcal DNA uptake sequences [70]. pGCC6 carries a chloramphenicol resistance cassette and a *lac* promoter/operator at an intergenic chromosomal site located between the gonococcal genes *lctP* and *aspC*. Cloning in front of the *lac* promoter thus allows for chromosomal integration of the insert between *lctP* and *aspC* in *N. gonorrhoeae* and subsequent IPTG inducible expression [71]. Isolation and purification of plasmid DNA was performed using QIAprep Spin Miniprep columns according to manufacturers specifications (Qiagen, Chatsworth, CA, U.S.A). The nucleotide sequences of all clones and constructs were determined from plasmid DNA or from PCR products by ABI 3730 high-throughput capillary electrophoresis sequencing.

**Table 1:** *Neisseria* strains used in this work.

Strain name	Parental strain	Relevant genotype	Reference
VD300	MS11	Opa^−^ derivative of MS11	[79]
KS101	VD300	*pilE_ind_*	[70]
KS335	VD300	*ccoP*_2x_ a	This study
KS336	VD300	*cycB::tet*	This study
KS337	KS335	*ccoP*_2x_ a, *cycB::tet*	This study
KS338	KS335	*ccoP_Ngo_* b	This study
KS339	KS337	*ccoP_Ngo_* b, *cycB::tet*	This study
KS340	VD300	*ccoP_Nme_* c	This study
KS341	KS340	*ccoP_Nme_* c, *cycB::tet*	This study
KS342	KS336	*cycB::tet*, *iga::cycB*	This study
KS343	KS337	*ccoP*_2x_ a, *cycB::tet*, *iga::cycB*	This study
KS344	KS101	*ccoP::pUP6::ccoP* d	This study
KS351	KS101	*ccoP*_2x_ a	This study
KS345	KS351	*ccoP*_2x_ a, *lctP::ccoP_ind_*	This study
KS346	KS345	*ccoP*_2x_::mTn*erm*26^e^, *lctP::ccoP_ind_*	This study
KS347	KS337	*ccoP*_2x_ a, *cycB::tet*, *iga::cycB-ccoP*	This study
MC58		Wild-type serogroup B strain	[80]
*c*_5_-	MC58	*cycB::tet*	[51]
KS348	MC58	*ccoP_Ngo_* b	This study
KS349	*c*_5_-	*ccoP_Ngo_* b, *cycB::tet*	This study
KS350	MC58	*lctP::ccoP*	This study

a) Di-heme form of *N. gonorrhoeae* CcoP made by changing codon number 362 from CAG to the stop codon TAG.

b) Di-heme form of *ccoP* exchanged by wildtype (tri-heme) *N. gonorrhoeae ccoP* by using pUP6NGO*ccoP*::mTn*erm*22 carrying a transposon insertion just downstream of the endogenous stop codon.

c) *N. gonorrhoeae ccoP* exchanged by MC58 *ccoP* by using pUP6NME*ccoP*::mTn*erm*17 carrying a transposon insertion just downstream of the endogenous stop codon in MC58.

d) Tandem duplication of *ccoP* made by transformation with the plasmid pUP6NGO*ccoP* (Campbell-type integration).

e) The endogenous/wildtype *ccoP*_2x_ locus in *N. gonorrhoeae* carries a transposon insertion in the second heme domain inactivating the gene (see Figure 6B; transposon labelled with double asterisk).

### Nucleotide sequence determination of *ccoP* alleles

Amplification and sequencing of *ccoP* genes from strains described in Tables S1 and S2 were completed using the primers listed in Table S4. PCR amplification consisted of a denaturation step at 94°C for five minutes followed by 30 cycles of 94°C for 30 s, 55°C for 30 s, and 72°C for three minutes. PCR products were PEG purified and sequenced directly by cycle sequencing with BigDye Ready Reaction Mix (Applied Biosystems) according to manufacturer's instructions and using an ABI 377 automated DNA sequencer.

### Sequence data manipulation and analysis

Nucleotide sequence data for forward and reverse strands were assembled with the STADEN software package [72], reformatted into the Genetics Computer Group (GCG) format and aligned manually using the Molecular Evolutionary Genetics Analysis (MEGA) v4 software package [73]. Phylogenetic relationships between individual sequences were inferred using ClonalFrame v1.1 [74]. This software is based on a model of genetic diversification that accounts for the way recombination occurs in bacterial populations. This enables the inference of phylogenetic relationships based on sequence data even if they are partly incongruent due to recombination. Sequence data for all isolates were input into ClonalFrame and default values were used for all options. Three independent runs were performed, each consisting of 300,000 iterations with 100,000 burn-in iterations. The convergence of the Markov Chain Monte Carlo (MCMC) in the different runs were judged satisfactory based on the Gelman-Rubin test as implemented in ClonalFrame GUI interface. The samples from the three runs were then concatenated for further analysis with a 95% majority rule consensus tree constructed using the ClonalFrame GUI. The consensus tree was then imported as a newick file into MEGA v4 for further annotation. Calculations of recombination tests were performed using RDP3 [74].

### *ccoP* allele exchange between *N. gonorrhoeae* and *N. meningitidis*

To exchange the endogenous *ccoP* locus of *N. meningitidis* with the tri-heme *ccoP* allele of *N. gonorrhoeae* and vice versa, the endogenous *ccoP* locus of *N. gonorrhoeae* with the di-heme *ccoP* allele of *N. meningitidis*, the strains MC58 and VD300 were transformed with the plasmids pUP6NGO*ccoP*::mTn*erm#*22 and pUP6NME*ccoP*::mTn*erm#*17 respectively, and selected on GC agar plates containing erythromycin (each plasmid carries a transposon insertion downstream of the *ccoP* stop codon, see Figures 6A and B).

### Construction of a gonococcal strain expressing di-heme CcoP

The premature stop codon was introduced into *N. gonorrhoeae ccoP* by PCR-based splicing-by-overlap extension (SOE) reactions into an otheriwse wildtype allele. Each pair of PCR fragments containing the mutation was created using primers FE1141 (5′-CGGAATTCGAGCTCTCTTTATCTGTTTCCTGTTAGTAC-3′) in combination with FE1156 (5′-AACGGTTTC**GTAGAC** **CTA**TTTGCCATCCGCTTTGGCGGC-3′), and FE1155 (5′-CGGATGGCAAA**TAGGTCTAC**GAAACCGTTTGTGCCGCCTGCC-3′) in combination with Av2045 (5′-TTGGACGACGGACGAAGTCTC-3′) was used to make the second overlapping PCR fragment. Altered base pairs including the novel stop codon and an *Acc*I restriction site (underlined) are shown in bold. The overlapping PCR fragments were spliced together using primers *ccoP* seq5′ (5′- TCGGTTATCTGGTTATGTATCC -3′) and Av2044 (5′-GAATACGCTCTCCCTCTTTACC-3′). Purified PCR products were used to genetically transform *N. gonorrhoeae* strains [75] and correct transformants were screened for by *Acc*I digestion of PCR fragments. Direct DNA sequencing of PCR fragments derived from the transformants was done, using appropriate primer sets, to verify the introduction of the stop codon and the absence of any other alterations.

### Mini-transposon mutagenesis of the *ccoP* locus

The *N. gonorrhoeae ccoP* gene along with 133 bp upstream of the gene and 151 bp downstream of the gene was amplified using primers FE1140 (5′-TACTCTATATCGTCTTCAACAGG-3′) and FE1144 (5′-CAAAAATATCAGTCGGTCTGACTGC-3′). The resulting PCR fragments were digested with unique, flanking *EcoR*I/*Sac*I sites and cloned into the polylinker of pUP6, yielding plasmid pUP6NGO*ccoP*. Similarly, the *N. meningitidis ccoP* gene along with 133 bp upstream of the gene and 350 bp downstream of the gene was amplified using primers FE1140 and FE1181 (5′-GCGGGATCCGAGCTCTTACAACAAATAGGCAGTCTGCG-3′). The resulting PCR fragments was digested with unique, flanking *EcoR*I/*BamH*I and cloned into the polylinker of pUP6, yielding plasmid pUP6NME*ccoP*. Mini-transposon (mTn) mutagenesis was performed on both pUP6NGO*ccoP* and PUP6NME*ccoP* as previously described [76]. The series of mTn insertions, conferring resistance to either chloramphenicol (mTn*cm*) or erythromycin (mTn*erm*), were isolated, mapped by PCR and sequencing using appropriate primer sets, depending on the location of the transposon insertion. Primer sequences and the detailed location of each transposon insertion site are available upon request.

### Construction and expression of an ectopic de-repressible *ccoP* allele

For construction of a gonococcal strain carrying an ectopic de-repressible *ccoP* gene the coding region of *ccoP* and 20 bp of upstream DNA, to include the RBS, was amplified by PCR from *N. gonorrhoeae* strain VD300 by using the forward primer av2226 (5′ - CGAAAACCTTAATTAATGTGATAACGGAGCAAAACAATG - 3′), *Pac*I site underlined, and the reverse primer FE1144 (5′ - CAAAAATATCAGTCGGTCTGACTGC - 3′). The PCR reaction was performed by Advantage HD polymerase (AH diagnostics) to create blunt ends. The resulting PCR product was cut with *Pac*I and cloned into pGCC6, digested with *Pme*I and *Pac*I, at an intergenic chromosomal site located between the gonococcal genes *lctP* and *aspC* and linked to the *lac* promoter/operator. The resulting plasmid pGCC6NGO*ccoP* was then used to transform strain KS351 (*ccoP*_2x_) and transformants were selected for growth on GC agar plates containing chloramphenicol. To inactivate the *ccoP* allele from the endogenous locus, the *N. gonorrhoeae* strain carrying the ectopic de-repressible *ccoP* allele (KS345) was transformed with pUP6NGO*ccoP::*mTn*erm#*26 (position of this mTn insertion is labelled with two asterisks in Figure 6B) and selected on GC agar plates containing erythromycin and 250µM IPTG.

### Construction of *ccoP* transposon insertion mutants in *N. gonorrhoeae*

To create an *N. gonorrhoeae* strain carrying a tandem duplication of *ccoP*, strain KS101 was transformed with pUP6NGO*ccoP* and transformants were selected on GC agar plates containing kanamycin. PCR using appropriate primer sets was used to confirm the correct (Campbell-type) integration of pUP6NGO*ccoP* on the chromosome. The resulting *N. gonorrhoeae* strain carrying a tandem duplication of *ccoP* (KS344) was transformed with a series of characterized pUP6NGO*ccoP*::mTn*erm* and pUP6NGO*ccoP*::mTn*cm* plasmids and transformants were selected on GC plates containing erythromycin and chloramphenicol respectively. The location of each transposon insertion, to either the expressed or non-expressed copy of *ccoP*, was determined by PCR using appropriate primer sets (available upon request).

### Construction of *ccoP* transposon insertion mutants in *N. meningitidis*

For this purpose, the wildtype *N. meningitidis* strain MC58 was transformed with the plasmid pGCC6NGO*ccoP* to create a strain carrying an ectopic copy of *N. gonorrhoeae ccoP*. Transformants were selected on GC agar plates containing chloramphenicol and confirmed correct by appropriate PCR primer sets and immunoblotting followed by heme-staining to visualize expression of the tri-heme form of CcoP. The resulting *N. meningitidis* strain (KS350) carrying the ectopic *N. gonorrhoeae ccoP* and the wildtype strain MC58 were transformed in parallel with a series of characterized pUP6NME*ccoP*::mTn*erm* plasmids and transformants were selected on GC plates containing erythromycin.

### Construction of *cycB* mutants

To generate the *cycB* insertion mutants, [51] the plasmid *cycB*::tet was introduced into MC58 and into VD300 by transformation and transformants were selected for growth on GC agar plates containing tetracycline. To obtain a complete deletion of *cycB* in *N. gonorrhoeae* the primers FE2223 (5′ - TCCGCAAAGCGGTGGAAATG- 3′) and FE2220 (5′ – GTTTAAACTGTCGCGGAGTTGTTTCATTTG - 3′) were used to amplify an 800 bp fragment of genomic DNA upstream of the *cycB* gene and the primers FE2221 (5′-TGAAACAACTCCGCGACAGTTTAAACACTATATGGCAAACCAATCCGGTGC -3′) and FE2225 (5′ –TATTTTGACAAACCACCGGAG - 3′) were used to amplify a 900 bp fragment of genomic DNA downstream of the *cycB* gene. The PCR products contained regions of homology at the 3′ end of the upstream fragment and at the 5′ end of the downstream fragment such that they could be spliced together by PCR-based splicing-by-overlap extension leaving out the entire *cycB* gene. This was done by using the primers FE2224 (5′–TTCATCCGGACAAACGCGTTG -3′) and FE2222 (5′–AACCTGTCGCTCTACGGCGAAC- 3′). The purified PCR product was used to genetically transform *N. gonorrhoeae* by a non-selective transformation technique [75], and correct transformants were screened for by PCR using the primers FE2223 and FE2225. The absence of *c*_5_ expression, including the truncated *c*_5_ form seen in the *cycB*::tet mutants, was verified by heme-stained blots of whole cell extracts.

### Ectopic expression of *cycB* and a *cycB-ccoP* hybrid allele in *N. gonorrhoeae*

Ectopic expression of *cycB* and the *cycB-ccoP* hybrid allele was performed by cloning PCR amplified products into a unique *Sac*I restriction site in plasmid p2/16/1 [70], allowing integration into the *iga* locus of the gonococcal chromosome. The resulting plasmids were then used to transform the mutant KS337 (VD300 *ccoP*_2x_, *cycB*) and transformants were selected for growth on GC agar plates containing erythromycin.

For cloning of the wildtype *cycB* allele the coding region including about 250 bp of upstream DNA was amplified by PCR from *N. gonorrhoeae* strain VD300 by using the forward primer c555F_*Sac*I (5′ – TGCAGAGCTCAATTGGCAAAGGTTATCTTGCG - 3′) in combination with the reverse primer FE2200 (5′ – ATTCGAGCTCACACCCATTTGATGTCATTTCC - 3′), *Sac*I sites underlined.

The *cycB-ccoP* hybrid allele was constructed by PCR-based splicing-by-overlap extension such that the region encoding the amino-terminus of *cycB* encompassing the first *c*-type heme domain and about 250 bp of upstream DNA was fused to the region encoding the third heme domain of *ccoP*. The relative spacing between the two heme domains in the hybrid protein was maintained as seen for wildtype *cycB* by facilitating a conserved stretch of four residues (Ala-Ala-Pro-Ala) in the C-terminal end of the AlaSerPro-rich linker domains. The *cycB* part of the hybrid allele, including about 250 bp of upstream DNA, was amplified by PCR using the flanking primer c555F_*Sac*I in combination with the primer FE2216 (5′ – TCCGCTTTGGCCGCAGGGGCTGCCGCACCCTTGTC – 3′). The region encoding the third heme domain of *ccoP* was amplified by PCR using the flanking primer FE2218 (5′ – CCGGGAGCTCATTCGATATGAATCCGGATTTCTG – 3′), *Sac*I site underlined, in combination with the primer FE2217 (5′ – ACAAGGGTGCGGCCGCACCTGCCGCCAAAGCGGATG– 3′). The two overlapping PCR fragments were spliced together by using the flanking primers c555F_*Sac*I and FE2218.

### Nitrite consumption assays

Nitrite concentrations in culture media were measured by a colourimetric assay as previously detailed [51].

### Protein gels, immunoblotting and detection of *c*-type cytochromes

Whole-cell extracts for detection of *c*-type cytochromes were prepared by harvesting bacteria from plates into 10mM Hepes buffer pH 7.0, subjecting the suspension to 5 cycles of freezing and thawing, and finally suspending the bacteria in 1% w/v n-dodecyl β-D-maltoside (Sigma-Aldrich). After 10 min incubation at 50°C samples were separated on 10% or 12% Criterion XT Precast Gels (Bio-Rad Laboratories, Hercules CA, USA) and blotted onto PVDF membranes. To visualize heme-dependent, peroxidase activity of the *c*-type cytochromes, the membranes were first incubated with SuperSignal West Pico Chemiluminiscent Substrate, according to manufacturers instructions (PIERCE, Rockford, IL. USA), and then exposed to X-ray film (GE Healthcare, Buckinghamshire, UK) [77]. Immunoblotting was used to detect expression of nitrite reductase NirK. For SDS-PAGE, whole cell extracts were prepared from equivalent numbers of cells by heating cell suspensions to 100°C for 3 min in SDS-sample loading buffer. The primary antibody used to detect NirK was a polyclonal rabbit antibody and was diluted 1∶1000 [51]. Procedures for immunoblotting using alkaline phosphatase-coupled goat anti-rabbit and goat anti-mouse antibodies have been described previously [76].

## Supporting Information

Figure S1 CcoP polymorphisms. Distribution of polymorphisms along the 1, 336 nt of gene *ccoP* in the 33 alleles among *Neisseria* species. The scale above the graph is in amino acids. CcoP functional domains are represented as distinct blocks with green blocks representing heme groups and the red bar depicting the AlaSerPro-rich region. Black vertical bars above this represent synonymous nucleotide polymorphisms with non-synonymous polymorphisms depicted below the diagram. Red vertical bars both above and below represent synonymous and non-synonymous polymorphisms respectively detected among *N. gonorrhoeae*, *N. lactamica* and *N. meningitidis* isolates only. The asterisk (*) indicates the position of the stop codon found among *N. meningitidis* isolates. The vertical bars found beside to the left of the alignment beside allele numbers indicate the species to which the *ccoP* alleles belong; black: *N. gonorrhoeae*; red: *N. lactamica*; blue: *N. meningitidis*; green: other *Neisseria* species including *N. cinerea*, *N. polysaccharea*, *N. subflava*, *N. mucosa*, *N. flavescens* and *N. sicca*.
(7.54 MB TIF)

Figure S2 Effects of CcoP domain alterations on aerobic and microaerobic growth in *N. meningitidis*. Cultures of wild-type (MC58) (open squares); *ccoP_Ngo_* (KS348) (open circles); *cycB* (*c*_5_-) (filled squares) and *ccoP_Ngo_*, *cycB* (KS349) (filled circles) growing under aerobic conditions (A) and under microaerobic conditions without nitrite (B). The results shown are representative of three independent experiments.
(0.10 MB TIF)

Figure S3 Effects of CcoP domain alterations on aerobic and microaerobic growth in *N. gonorrhoeae*. Cultures of wild-type (VD300) (open squares); and mutants *ccoP*_2x_ (KS335) (open circles); *cycB* (KS336) (filled squares); *ccoP*_2x_, *cycB* (KS337) (filled circles); *ccoP_Nme_* (KS340) (open triangles); *ccoP_Nme_*, *cycB* (KS341) (filled triangles) growing under aerobic conditions (A) and under microaerobic conditions without nitrite (B). The results shown are representative of three independent experiments.
(0.09 MB TIF)

Figure S4 The *cycB* insertion mutant expresses a truncated *c*_5_ protein. The *cycB* gene, encoding the *c*_5_ protein, was disrupted by insertion of a tetracycline resistance (Tet^R^) gene [51]. The arrow indicates the ensuing *c*_5_ ORF that terminates at residue 171 followed by eleven residues derived from sequences within the tetracycline resistance gene insertion before a stop codon (indicated by an asterisk). This results in a 182 residue *c*_5_ protein retaining the membrane-proximal heme domain (in gray).
(0.05 MB TIF)

Figure S5 NirK expression during growth under microaerobic conditions. *N. gonorrhoeae* strains: 1, wild-type (VD300) and mutants 2, *cycB* (KS336); 3, *ccoP*_2x_ (KS335); 4, *ccoP*_2x_, *cycB* (KS337); 5, *ccoP_Nme_*(KS340); 6, *ccoP_Nme_*, *cycB* (KS341); were grown under microaerobic conditions plus 5 mM nitrite. Samples were taken after one (1) and six (6) hours of growth, and whole cell lysates were analyzed by immunoblotting with anti-NirK antibodies.
(0.15 MB TIF)

Figure S6 Strategy for construction of a *c*_5_-CcoP translational fusion. A translational fusion consisting of the amino-terminus of *c*_5_, encompassing the first *c*-type heme domain, and the third *c*-type heme domain of CcoP was made by exploiting a conserved stretch of residues (Ala-Ala-Pro-Ala, underlined) in the AlaSerPro-rich linker domains. Also underlined are the two cysteine and single histidine residues found in a CXXCH motif required for disfulfide bonding to the vinyl groups of heme. The total number of amino acids (and thus the relative spacing) between the two *c*-type heme domains was maintained as seen for wildtype *c*_5_. The hybrid-encoding gene was then expressed from an ectopic site.
(15.81 MB TIF)

Table S1 (0.15 MB DOC)

Table S2 (0.09 MB DOC)

Table S3 (0.04 MB DOC)

Table S4 (0.03 MB DOC)
