# Supplementary figures and images for: Structural Alterations in a Component of Cytochrome c Oxidase and Molecular Evolution of Pathogenic Neisseria in Humans
Source: PLoS Pathog. 2010 Aug 19;6(8):e1001055. doi: 10.1371/journal.ppat.1001055 (PMC2924362; doi:10.1371/journal.ppat.1001055)

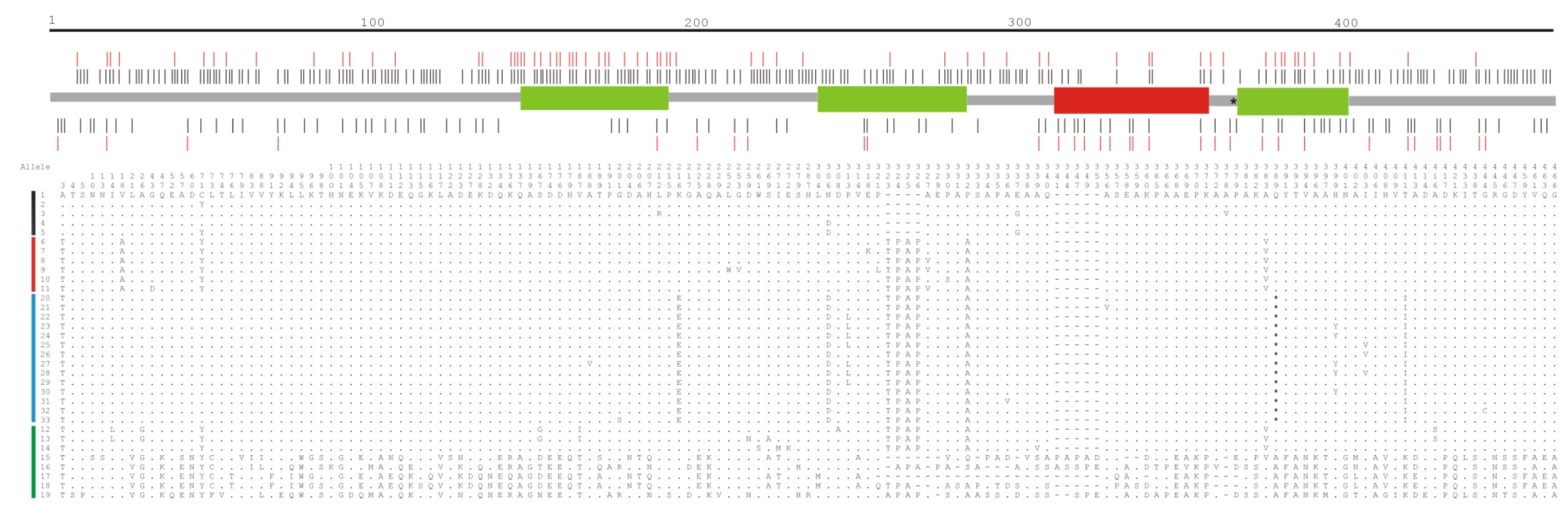

Supplement: Figure S1 — CcoP polymorphisms. Distribution of polymorphisms along the 1, 336 nt of gene ccoP in the 33 alleles among Neisseria species. The scale above the graph is in amino acids. CcoP functional domains are represented as distinct blocks with green blocks representing heme groups and the red bar depicting the AlaSerPro-rich region. Black vertical bars above this represent synonymous nucleotide polymorphisms with non-synonymous polymorphisms depicted below the diagram. Red vertical bars both above and below represent synonymous and non-synonymous polymorphisms respectively detected among N. gonorrhoeae, N. lactamica and N. meningitidis isolates only. The asterisk (*) indicates the position of the stop codon found among N. meningitidis isolates. The vertical bars found beside to the left of the alignment beside allele numbers indicate the species to which the ccoP alleles belong; black: N. gonorrhoeae; red: N. lactamica; blue: N. meningitidis; green: other Neisseria species including N. cinerea, N. polysaccharea, N. subflava, N. mucosa, N. flavescens and N. sicca. (7.54 MB TIF) [file ppat.1001055.s001.tif]

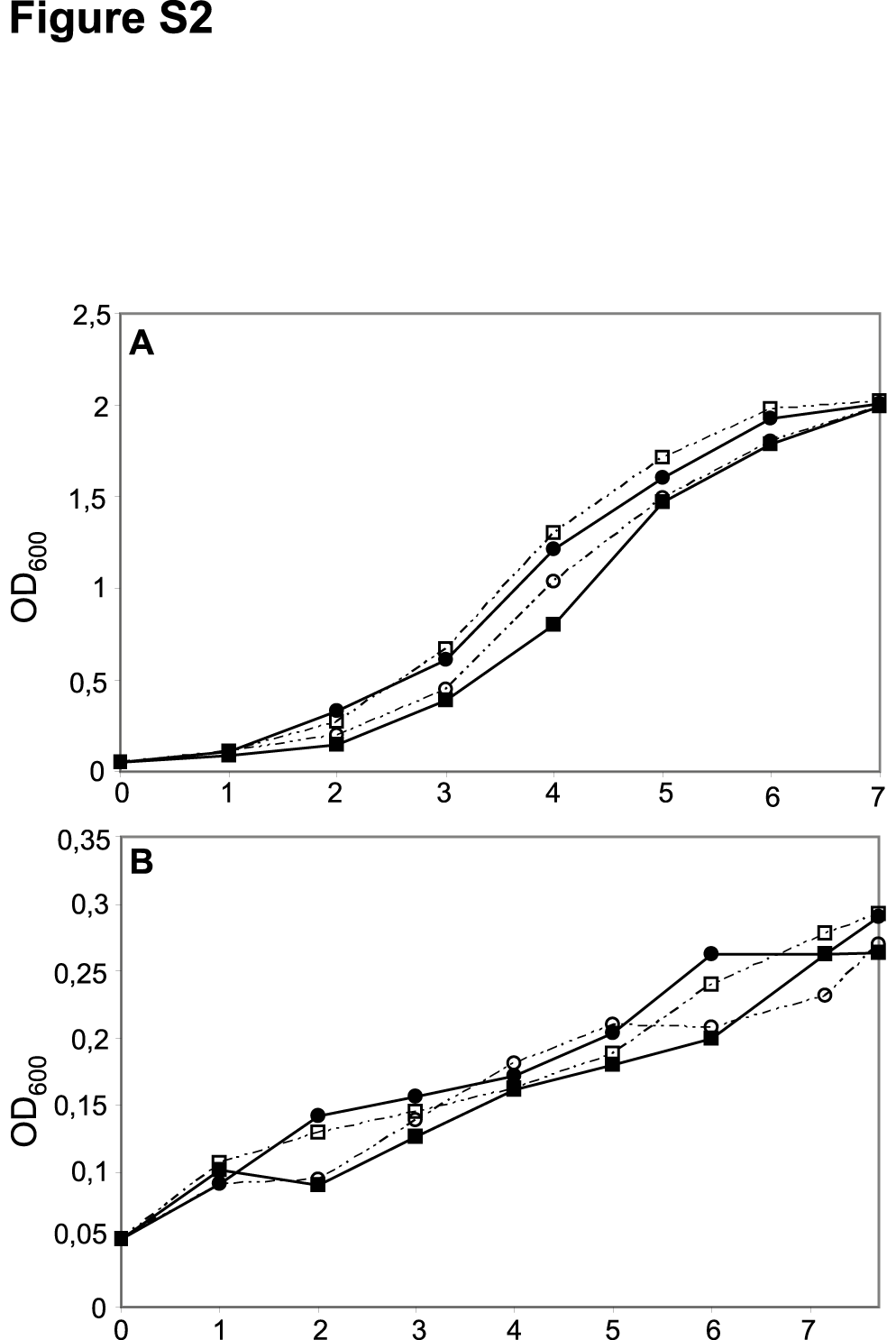

Supplement: Figure S2 — Effects of CcoP domain alterations on aerobic and microaerobic growth in N. meningitidis. Cultures of wild-type (MC58) (open squares); ccoPNgo (KS348) (open circles); cycB (c 5-) (filled squares) and ccoPNgo, cycB (KS349) (filled circles) growing under aerobic conditions (A) and under microaerobic conditions without nitrite (B). The results shown are representative of three independent experiments. (0.10 MB TIF) [file ppat.1001055.s002.tif]

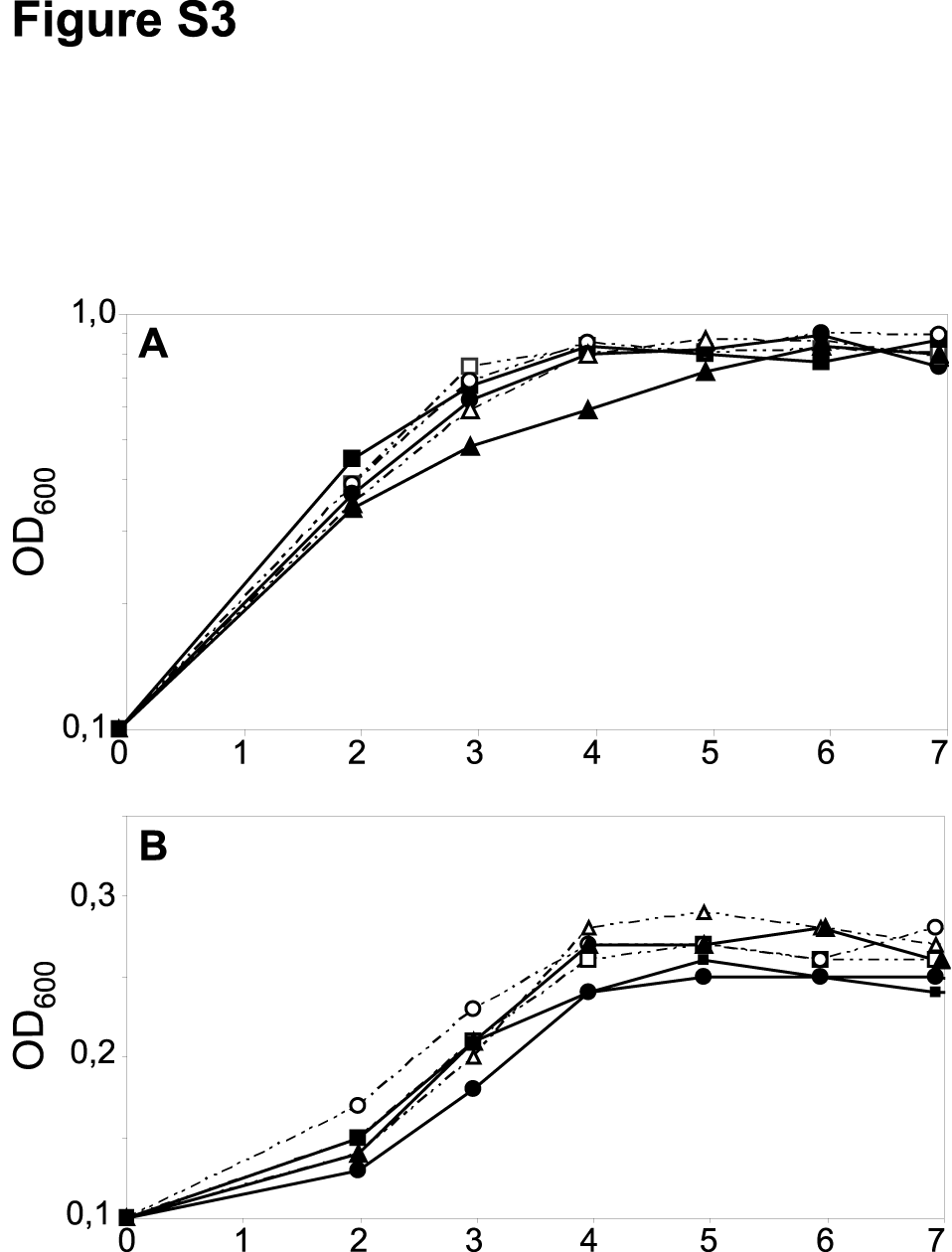

Supplement: Figure S3 — Effects of CcoP domain alterations on aerobic and microaerobic growth in N. gonorrhoeae. Cultures of wild-type (VD300) (open squares); and mutants ccoP 2x (KS335) (open circles); cycB (KS336) (filled squares); ccoP 2x, cycB (KS337) (filled circles); ccoPNme (KS340) (open triangles); ccoPNme, cycB (KS341) (filled triangles) growing under aerobic conditions (A) and under microaerobic conditions without nitrite (B). The results shown are representative of three independent experiments. (0.09 MB TIF) [file ppat.1001055.s003.tif]

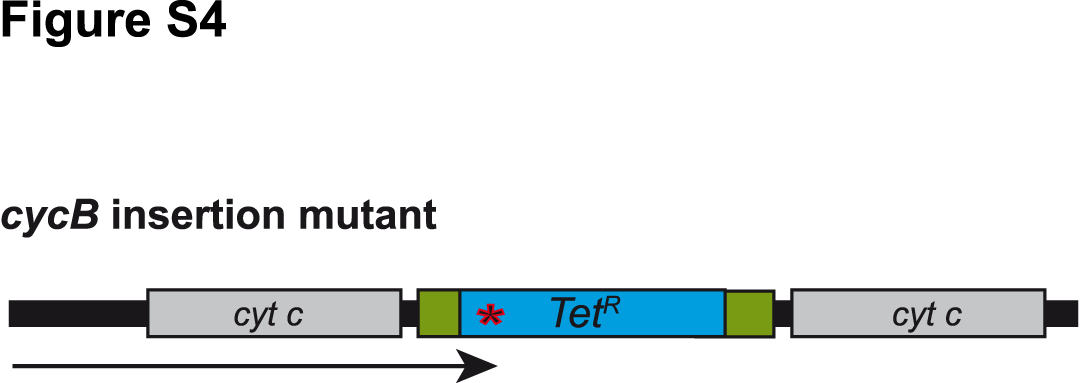

Supplement: Figure S4 — The cycB insertion mutant expresses a truncated c 5 protein. The cycB gene, encoding the c 5 protein, was disrupted by insertion of a tetracycline resistance (TetR) gene [51]. The arrow indicates the ensuing c 5 ORF that terminates at residue 171 followed by eleven residues derived from sequences within the tetracycline resistance gene insertion before a stop codon (indicated by an asterisk). This results in a 182 residue c 5 protein retaining the membrane-proximal heme domain (in gray). (0.05 MB TIF) [file ppat.1001055.s004.tif]

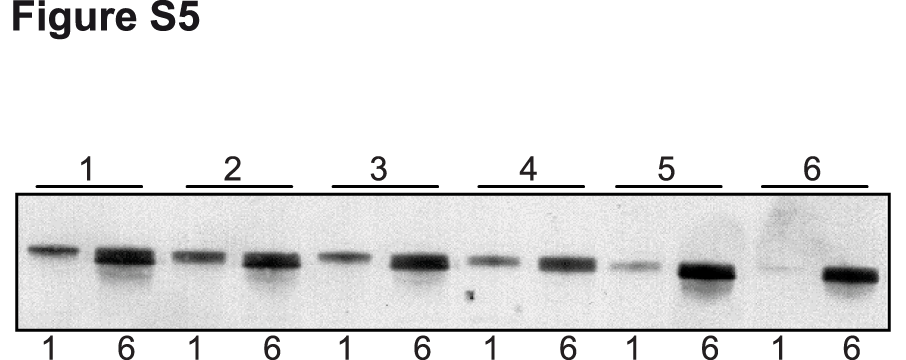

Supplement: Figure S5 — NirK expression during growth under microaerobic conditions. N. gonorrhoeae strains: 1, wild-type (VD300) and mutants 2, cycB (KS336); 3, ccoP 2x (KS335); 4, ccoP 2x, cycB (KS337); 5, ccoPNme(KS340); 6, ccoPNme, cycB (KS341); were grown under microaerobic conditions plus 5 mM nitrite. Samples were taken after one (1) and six (6) hours of growth, and whole cell lysates were analyzed by immunoblotting with anti-NirK antibodies. (0.15 MB TIF) [file ppat.1001055.s005.tif]

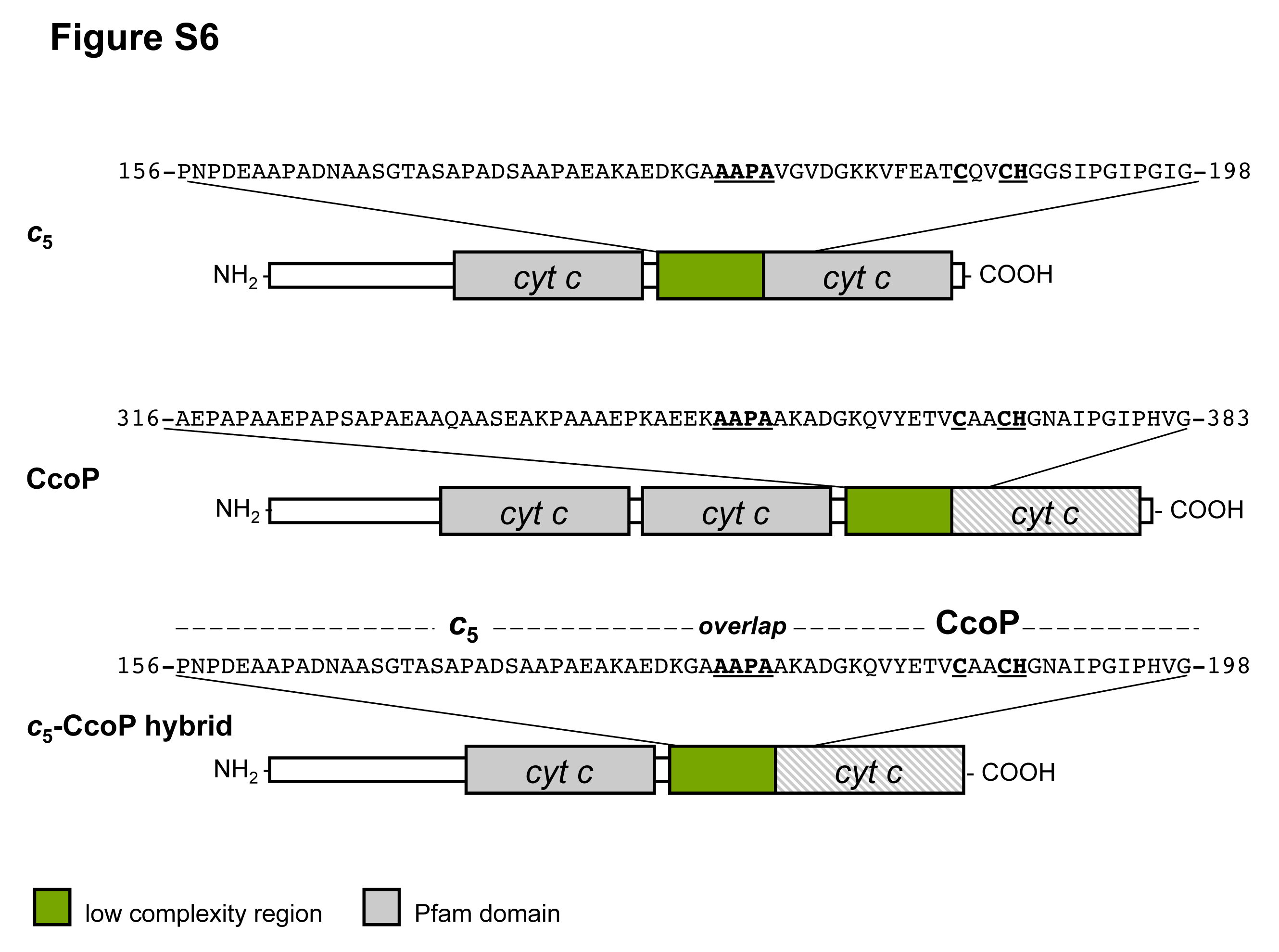

Supplement: Figure S6 — Strategy for construction of a c 5-CcoP translational fusion. A translational fusion consisting of the amino-terminus of c 5, encompassing the first c-type heme domain, and the third c-type heme domain of CcoP was made by exploiting a conserved stretch of residues (Ala-Ala-Pro-Ala, underlined) in the AlaSerPro-rich linker domains. Also underlined are the two cysteine and single histidine residues found in a CXXCH motif required for disfulfide bonding to the vinyl groups of heme. The total number of amino acids (and thus the relative spacing) between the two c-type heme domains was maintained as seen for wildtype c 5. The hybrid-encoding gene was then expressed from an ectopic site. (15.81 MB TIF) [file ppat.1001055.s006.tif]
