# Supplementary material for: Structural Alterations in a Component of Cytochrome c Oxidase and Molecular Evolution of Pathogenic Neisseria in Humans
Source: PLoS Pathog. 2010 Aug 19;6(8):e1001055. doi: 10.1371/journal.ppat.1001055 (PMC2924362; doi:10.1371/journal.ppat.1001055)
Supplement: Table S1 — (0.15 MB DOC) [file ppat.1001055.s007.doc]

**Supplementary Table S1. *N. meningitidis*** strains used in this study

| ***N. meningitidis*** | **ST and Clonal Complex** | **Serogroup** | **Country of origin** | **Disease** | ***ccoP* allele** | **Genome accession No.** | **Genbank accession #** |
| --- | --- | --- | --- | --- | --- | --- | --- |
| Z2491 | ST-4 (cc4) | A | Gambia | invasive | 1 | AL157959^a^ | HM543190 |
| MC58 | ST-74 (cc32) | B | England | invasive | 9 | AE002098^b^ | HM543198 |
| FAM18 | ST-11 (cc11) | C | unknown | invasive | 6 | AM421808^a^ | HM543206 |
| 053442 | ST-4821 (cc4821) | C | China | invasive | 10 | CP000381^c^ | HM584893 |
| alpha14 | ST-53 (cc53) | cps null | Germany | carrier | 11 | AM889136 | HM584892 |
| 6748 | ST-1 (cc1) | A | Canada | invasive | 6 | - | HM543215 |
| 106 | ST-1 (cc1) | A | Morocco | invasive | 6 | - | HM543216 |
| 322/85 | ST-2 (cc1) | A | East Germany | invasive | 6 | - | HM543217 |
| 20 | ST-1 (cc1) | A | Niger | invasive | 49 | - | HM543257 |
| 371 | ST-1 (cc1) | A | India | invasive | 6 | - | HM543218 |
| 254 | ST-1 (cc1) | A | Djibouti | invasive | 49 | - | HM543258 |
| 120M | ST-1 (cc1) | A | Pakistan | invasive | 6 | - | HM543219 |
| 393 | ST-1 (cc1) | A | Greece | carrier | 6 | - | HM543220 |
| BZ133 | ST-1 (cc1) | B | Netherlands | invasive | 6 | - | HM543221 |
| 129E | ST-1 (cc1) | A | West Germany | invasive | 6 | - | HM543222 |
| 79128 | ST-3 (cc1) | A | China | invasive | 6 | - | HM543223 |
| D8 | ST-4 (cc4) | A | Mali | invasive | 1 | - | HM543192 |
| 10 | ST-4 (cc4) | A | Burkina Faso | invasive | 1 | - | HM543194 |
| 2059001 | ST-4 (cc4) | A | Mali | invasive | 1 | - | HM543191 |
| 690 | ST-4 (cc4) | A | India | invasive | 1 | - | HM543193 |
| 255 | ST-4 (cc4) | A | Burkina Faso | invasive | 1 | - | HM543197 |
| S3131 | ST-4 (cc4) | A | Ghana | invasive | 1 | - | HM543195 |
| 1014 | ST-4 (cc4) | A | Sudan | invasive | 1 | - | HM543196 |
| 14/1455 | ST-5 (cc5) | A | Russia | unspecified | 24 | - | HM543236 |
| F6124 | ST-5 (cc5) | A | Chad | invasive | 24 | - | HM543237 |
| F4698 | ST-5 (cc5) | A | Saudi | carrier | 24 | - | HM543238 |
| 7891 | ST-5 (cc5) | A | Finland | invasive | 24 | - | HM543239 |
| H1964 | ST-5 (cc5) | A | UK | invasive | 24 | - | HM543240 |

| ***N. meningitidis*** | **ST and Clonal Complex** | **Serogroup** | **Country of origin** | **Disease** | ***ccoP* allele** | **Genome accession No.** | **Genbank accession #** |
| --- | --- | --- | --- | --- | --- | --- | --- |
| IAL2229 | ST-5 (cc5) | A | Brazil | unspecified | 24 |  | HM543241 |
| 11-004 | ST-5 (cc5) | A | China | invasive | 24 |  | HM543242 |
| 153 | ST-5 (cc5) | A | China | invasive | 24 | - | HM543243 |
| BZ10 | ST-8 (cc8) | B | Netherlands | invasive | 26 | - | HM543245 |
| B6116/77 | ST-10 (cc8) | B | Iceland | invasive | 26 | - | HM543246 |
| G2136 | ST-8 (cc8) | B | England | invasive | 26 | - | HM543247 |
| 94/155 | ST-66 (cc8) | C | New Zealand | invasive | 26 | - | HM543248 |
| AK22 | ST-8 (cc8) | B | Greece | invasive | 26 | - | HM543249 |
| NGP20 | ST-11 (cc11) | B | Norway | invasive | 6 | - | HM543208 |
| 90/18311 | ST-11 (cc11) | C | Scotland | unspecified | 6 | - | HM543209 |
| L93/4286 | ST-11 (cc11) | C | England | invasive | 6 | - | HM543210 |
| D1 | ST-11 (cc11) | C | Mali | carrier | 6 | - | HM543211 |
| 38VI | ST-11 (cc11) | B | USA | unspecified | 6 | - | HM543212 |
| F1576 | ST-11 (cc11) | C | Ghana | unspecified | 6 | - | HM543213 |
| 500 | ST-11 (cc11) | C | Italy | unspecified | 6 | - | HM543207 |
| 1000 | ST-20 (cc18) | B | Russia | invasive | 22 | - | HM543250 |
| EG328 | ST-18 (cc18) | B | Germany | invasive | 25 | - | HM543251 |
| A22 | ST-22 (cc22) | W135 | Norway | carrier | 6 | - | HM543224 |
| 71/94 | ST-23 (cc23) | Y | Norway | invasive | 6 | - | HM543225 |
| NGPB24 | ST-32 (cc32) | B | Norway | invasive | 9 | - | HM543201 |
| 44/76 | ST-32 (cc32) | B | Norway | invasive | 9 | - | HM543199 |
| BZ169 | ST-32 (cc32) | B | Netherlands | invasive | 9 | - | HM543200 |
| EG329 | ST-32 (cc32) | B | East Germany | invasive | 9 | - | HM543203 |
| 196/87 | ST-32 (cc32) | C | Norway | unspecified | 9 | - | HM543204 |
| 204/92 | ST-33 (cc32) | B | Cuba | invasive | 9 | - | HM543202 |
| BZ83 | ST-34 (cc32) | B | Netherlands | invasive | 9 | - | HM543205 |
| 50/94 | ST-45 (cc41/44) | B | Norway | invasive | 19 | - | HM543226 |
| BZ198 | ST-41 (cc41/44) | B | Netherlands | invasive | 19 | - | HM543227 |
| ***N. meningitidis*** | **ST and Clonal Complex** | **Serogroup** | **Country of origin** | **disease** | ***ccoP* allele** | **Genome accession No.** | **Genbank accession #** |
| NGH15 | ST-43 (cc41/44) | B | Norway | carrier | 19 | **-** | HM543228 |
| 400 | ST-40 (cc41/44) | B | Austria | invasive | 19 | **-** | HM543229 |
| 88/06415 | ST-46 (cc41/44) | B | Scotland | invasive | 19 | **-** | HM543230 |
| 931905 | ST-41 (cc41/44) | B | Netherlands | invasive | 19 | **-** | HM543231 |
| 91/40 | ST-42 (cc41/44) | B | New Zealand | invasive | 19 | **-** | HM543232 |
| NGH36 | ST-47 (cc41/44) | B | Norway | carrier | 19 | - | HM543233 |
| NG3/88 | ST-12 (cc-) | B | Norway | invasive | 52 | - | HM543259 |
| EG011 | ST-36 (cc-) | B | East Germany | invasive | 51 | - | HM543261 |
| NGE28 | ST-26 (cc-) | B | Norway | carrier | 54 | - | HM543262 |
| NG4/88 | ST-30 (cc-) | B | Norway | invasive | 6 | - | HM543214 |
| NGH38 | ST-36 (cc-) | B | Norway | carrier | 24 | - | HM543244 |
| 3906 | ST-17 (cc-) | B | China | invasive | 27 | - | HM543255 |
| 860060 | ST-24 (cc750) | X | Netherlands | invasive | 29 | - | HM543256 |
| CN100 | ST-21 (cc-) | A | England | invasive | 50 | - | HM543263 |
| NG6/88 | ST-13 (cc269) | B | Norway | invasive | 52 | - | HM543260 |
| OX9930033 | ST-53 (cc53) | cps null | England | carrier | 55 | - | HM584884 |
| OX9930134 | ST-39 (cc198) | cps null | England | carrier | 28 | - | HM543252 |
| OX9930148 | ST-1454 (cc334) | cps null | England | carrier | 56 | - | HM584888 |
| OX9930150 | ST-1642 (cc-) | cps null | England | carrier | 56 | - | HM584889 |
| OX9930244 | ST-1452 (cc53) | cps null | England | carrier | 55 | - | HM584885 |
| OX9930254 | ST-198 (cc198) | cps null | England | carrier | 28 | - | HM543253 |
| OX9930310 | ST-198 (cc198) | cps null | England | carrier | 28 | - | HM543254 |
| OX9930314 | ST-53 (cc53) | cps null | England | carrier | 55 | - | HM584886 |
| OX9930654 | ST-1428 (cc-) | cps null | England | carrier | 57 | - | HM584890 |
| OX9930685 | ST-1654 (cc53) | cps null | England | carrier | 55 | - | HM584887 |

Genome sequencing projects with publically available genomic data:

^a^ The Sanger Centre

^b^ J. Craig Venter Institute, USA

^c^ Microbial Genome Centre of Chinese Ministry of Health
