# Supplementary material for: Structural Alterations in a Component of Cytochrome c Oxidase and Molecular Evolution of Pathogenic Neisseria in Humans
Source: PLoS Pathog. 2010 Aug 19;6(8):e1001055. doi: 10.1371/journal.ppat.1001055 (PMC2924362; doi:10.1371/journal.ppat.1001055)
Supplement: Table S2 — (0.09 MB DOC) [file ppat.1001055.s008.doc]

**Supplementary Table S2. *N. gonorrhoeae*, *N. lactamica* and commensal neisserial strains used in this study**

| **Isolate** | **ST and clonal complex** | **Site of isolation** | **Country of origin** | ***ccoP* allele** | **Genome Accession No** | **Genbank accession #** |
| --- | --- | --- | --- | --- | --- | --- |
| *N. gonorrhoeae* FA1090 | ST-1899 | DGI | USA | 5 | AE004969 | HM460521 |
| *N. gonorrhoeae* FA19 | ST-1892 | DGI | USA | 2 | ABZJ01000098^a^ | HM460529 |
| *N. gonorrhoeae* F62 | ST-1900 | genitourinary | USA | 31 | ADAA010000739 | HM460535 |
| *N. gonorrhoeae* 29528 | ST-1585 | genitourinary | UK | 32 | - | HM460536 |
| *N. gonorrhoeae* 29214 | ST-1895 | genitourinary | China | 5 | - | HM460522 |
| *N. gonorrhoeae* 28839 | ST-1594 | genitourinary | UK | 2 | - | HM460530 |
| *N. gonorrhoeae* 27921 | ST-1905 | genitourinary | Uzbekistan | 5 | - | HM460523 |
| *N. gonorrhoeae* 27806 | ST-1892 | DGI | UK | 2 | - | HM460531 |
| *N. gonorrhoeae* 26034 | ST-1906 | DGI | unknown | 36 | - | HM460544 |
| *N. gonorrhoeae* 25562 | ST-1893 | DGI | unknown | 33 | - | HM460537 |
| *N. gonorrhoeae* 22584 | ST-1579 | genitourinary | USA | 34 | - | HM460538 |
| *N. gonorrhoeae* DGI2 | - | DGI | USA | 2 | ACIG01000046^a^ | HM460532 |
| *N. gonorrhoeae* 1291 | - |  | unknown | 35 | ABZF01000110^a^ | HM460539 |
| *N. gonorrhoeae* 35/02 | - |  | unknown | 5 | ABZG01000106^a^ | HM460524 |
| *N. gonorrhoeae* DGI18 | - | DGI | unknown | 5 | ABZH01000101^a^ | HM460525 |
| *N. gonorrhoeae* FA6140 | - |  | unknown | 3 | ABZI01000105^a^ | HM460540 |
| *N. gonorrhoeae* MS11 | ST-6959 | genitourinary | USA | 4 | ABZK01000106^a^ | HM460542 |
| *N. gonorrhoeae* PID18 | - | PID | unknown | 2 | ABZL01000114^a^ | HM460533 |
| *N. gonorrhoeae* PID1 | - | PID | unknown | 3 | ABZM01000093^a^ | HM460541 |
| *N. gonorrhoeae* PID24-1 | - | PID | unknown | 5 | ABZN01000079^a^ | HM460526 |
| *N. gonorrhoeae* PID332 | - | PID | unknown | 2 | ABZO01000115^a^ | HM460534 |
| *N. gonorrhoeae* SK-92-679 | - |  | unknown | 5 | ABZP01000132^a^ | HM460527 |
| *N. gonorrhoeae* SK-93-1035 | - |  | unknown | 36 | ABZQ01000106^a^ | HM460543 |
| *N. gonorrhoeae* NCCP11945 | ST-1901 | vaginal smear | South Korea | 5 | CP001050 | HM460528 |
| *N. lactamica* ATCC 23970 | ST-3787 | nasopharynx | unknown | 7 | ACEQ02000001^b^ | HM460545 |
| *N. lactamica* 020/06 | ST-640 | nasopharynx | UK | 8 | not available^c^ | HM460546 |
| **Isolate** | **ST and clonal complex** | **Site of isolation** | **Country of origin** | ***ccoP* allele** | **Genome Accession No** | **Genbank accession #** |
| *N. lactamica* 012/12 | ST-631 | nasopharynx | UK | 38 | - | HM460548 |
| *N. lactamica* 014/24 | ST-586 | nasopharynx | UK | 39 | - | HM460549 |
| *N. lactamica* 016/24 | ST-594 | nasopharynx | UK | 40 | - | HM460550 |
| *N. lactamica* 028/12 | ST-584 | nasopharynx | UK | 41 | - | HM460551 |
| *N. lactamica* 030/24 | ST-585 | nasopharynx | UK | 42 | - | HM460553 |
| *N. lactamica* 039/03 | ST-582 | nasopharynx | UK | 41 | - | HM460552 |
| *N. lactamica* 066/10 | ST-583 (cc1494) | nasopharynx | UK | 43 | - | HM460554 |
| *N. lactamica* 081/24 | ST-633 | nasopharynx | UK | 44 | - | HM460555 |
| *N. lactamica* 090/10 | ST-603 | nasopharynx | UK | 45 | - | HM460556 |
| *N. lactamica* 094/24 | ST-590 | nasopharynx | UK | 46 | - | HM460557 |
| *N. cinerea* ATCC14685 | ST-3579 | nasopharynx | Germany | 16 | ACDY02000012^b^ | HM460513 |
| *N. cinerea* LNP415 | ST-3579 | nasopharynx | Germany | 47 | - | HM460512 |
| *N. polysaccharea* 90400 | - | nasopharynx | Canada | 48 | - | HM460514 |
| *N. polysaccharea* ATCC43768 | ST-3557 | nasopharynx | unknown | 23 | ADBE01000060 ^b^ | HM460515 |
| *N. subflava* NJ9703 | - | oral cavity | unknown | 18 | ACEO02000003^b^ | HM460516 |
| *N. mucosa* ATCC25996 | ST-8082 | nasopharynx | unknown | 17 | ACDX02000003^b^ | HM460517 |
| *N. flavescens* NRL30031 | ST-3576 | nasopharynx | USA | 14 | ACEN01000073^b^ | HM460519 |
| *N. flavescens* SK114 | - | human skin | USA | 15 | ACQV01000016^d^ | HM460518 |
| *N. sicca* ATCC29256 | ST-8081 | pharyngeal mucosa | USA | 13 | ACKO02000008^c^ | HM460520 |
| *Neisseria sp. oral taxon* 014 str. F0314 | - | unknown | USA | 58 | ADEA01000029 ^a^ |  |

Genome sequencing projects with publically available genomic data:

^a^ The Broad Institute Genome Sequencing Platform, USA

^b^ Genome Sequencing Center at the Washington University, USA

^c^ The Sanger Centre

^d^ J. Craig Venter Institute, USA
