# Supplementary material for: Structural Alterations in a Component of Cytochrome c Oxidase and Molecular Evolution of Pathogenic Neisseria in Humans
Source: PLoS Pathog. 2010 Aug 19;6(8):e1001055. doi: 10.1371/journal.ppat.1001055 (PMC2924362; doi:10.1371/journal.ppat.1001055)
Supplement: Table S3 — (0.04 MB DOC) [file ppat.1001055.s009.doc]

| **Supplementary Table S3.** Recombination events detected in *ccoP* using RDP3 v3.41. | | | | |
| --- | --- | --- | --- | --- |
| **Event nr.** | **Receptor isolates** | **Donor isolates** | **Significance by method** | **Genomic position (bp)** |
| 1 | *N. cinerea* LNP415 | *N. cinerea* ATCC14685 | Genconv 7.831e-06 | 639-903 |
|  |  | *N. lactamica* 090/10 | BootScan 4.891e-07 |  |
|  |  |  | SiScan |  |
|  |  |  | 3Seq 9.236e-05 |  |
|  |  |  |  |  |
| 2 | *N. cinerea* ATCC14685 | *N. gonorrhoeae* 25562 | MaxChi 9.714e-03 | 869-1332 |
|  |  | *N.meningitidis* H1964 (ST-5) |  |  |
|  |  |  |  |  |
| 3 | *N. sicca* ATCC29256 | *N. flavescens* SK114 | MaxChi 5.857e-04 | 458-1106 |
|  |  | *N. subflava* NJ9703 | Chimaera 1.573e-04 |  |
|  |  |  | SiScan 4.696e-12 |  |
|  |  |  |  |  |
| 4 | *N. flavescens* SK114 | *N. sicca* ATCC29256 | MaxChi 1.055e-01 | 217-321 |
|  |  | *N. cinerea* LNP415 | SiScan 9.251e-04 |  |
|  |  |  |  |  |
| 5 | *N. flavescens* NRL30031 | *N.sicca* ATCC29256 | MaxChi 1.055e-01 | 191-321 |
|  |  | *N. cinerea* LNP415 | SiScan 9.251e-04 |  |
|  |  |  |  |  |
