# Supplementary material for: Structural Alterations in a Component of Cytochrome c Oxidase and Molecular Evolution of Pathogenic Neisseria in Humans
Source: PLoS Pathog. 2010 Aug 19;6(8):e1001055. doi: 10.1371/journal.ppat.1001055 (PMC2924362; doi:10.1371/journal.ppat.1001055)
Supplement: Table S4 — (0.03 MB DOC) [file ppat.1001055.s010.doc]

| **Primer** | **Primer base sequence (5’-3’)** | **Application** | **Location from 5’ end** |
| --- | --- | --- | --- |
| CcoP-P1F | CGGTATTCGYGCTCTTTTCAGG | *ccoP* forward primer | -183 (located within *ccoQ* subunit) |
| CcoP-P1R | CGGCTTCAGTTTTGATTTGTGTATCC | *ccoP* reverse primer | intergenic region |
| SEQ-FOR1 | CAAACTGTATGCCAAGTTTG | forward sequencing | 344 |
| SEQ-FOR2 | GACAAACTGCATATYATGAC | forward sequencing | 873 |
| SEQ-FOR3a | GAAATCCGGATTCATATC | forward sequencing *N. lactamica*, *N. gonorrhoeae, N. cinerea* and *N. polysaccharea* | 1360 |
| SEQ-FOR3b | GAAAATCCAGATTCAGTATTG | forward sequencing *N. meningitidis* | 1360 |
| SEQ-REV1 | CACAAACGGTTTCATAAAC | reverse sequencing | 1098 |
| SEQ-REV2 | GTTCYTCATCATACTG | reverse sequencing | 651 |
| SEQ-REV3 | CGTRGTTTGTACTTCTTC | reverse sequencing | 129 |

**Supplementary Table S4.** Primers used for amplification and sequencing of *ccoP* alleles.
